# The microRNA-29/PGC1α regulatory axis is critical for metabolic control of cardiac function

**DOI:** 10.1371/journal.pbio.2006247

**Published:** 2018-10-22

**Authors:** Xurde M. Caravia, Víctor Fanjul, Eduardo Oliver, David Roiz-Valle, Alba Morán-Álvarez, Gabriela Desdín-Micó, María Mittelbrunn, Roberto Cabo, José A. Vega, Francisco Rodríguez, Antonio Fueyo, Mónica Gómez, Manuel Lobo-González, Héctor Bueno, Gloria Velasco, José M. P. Freije, Vicente Andrés, Borja Ibáñez, Alejandro P. Ugalde, Carlos López-Otín

**Affiliations:** 1 Departamento de Bioquímica y Biología Molecular, Facultad de Medicina, Instituto Universitario de Oncología, Universidad de Oviedo, Oviedo, Spain; 2 Centro de Investigación Biomédica en Red de Cáncer (CIBERONC), Spain; 3 Centro Nacional de Investigaciones Cardiovasculares Carlos III (CNIC), Madrid, Spain; 4 Cardiology Department and Instituto de Investigación i+12, Hospital Universitario 12 de Octubre, Madrid, Spain; 5 Departamento de Morfología y Biología Celular, Facultad de Medicina, Universidad de Oviedo, Oviedo, Spain; 6 Facultad de Ciencias de la Salud, Universidad Autónoma de Chile, Chile; 7 Área de Fisiología, Departamento de Biología Funcional, Facultad de Medicina, Instituto Universitario de Oncología, Universidad de Oviedo, Oviedo, Spain; 8 Complejo Hospitalario Ruber Juan Bravo, Madrid, Spain; 9 Facultad de Medicina, Universidad Complutense de Madrid, Madrid, Spain; 10 Centro de Investigación Biomédica en Red de Enfermedades Cardiovasculares (CIBERCV), Spain; 11 IIS-Fundación Jiménez Díaz Hospital, Madrid, Spain; University of Pittsburgh, United States of America

## Abstract

Different microRNAs (miRNAs), including miR-29 family, may play a role in the development of heart failure (HF), but the underlying molecular mechanisms in HF pathogenesis remain unclear. We aimed at characterizing mice deficient in miR-29 in order to address the functional relevance of this family of miRNAs in the cardiovascular system and its contribution to heart disease. In this work, we show that mice deficient in *miR-29a/b1* develop vascular remodeling and systemic hypertension, as well as HF with preserved ejection fraction (HFpEF) characterized by myocardial fibrosis, diastolic dysfunction, and pulmonary congestion, and die prematurely. We also found evidence that the absence of miR-29 triggers the up-regulation of its target, the master metabolic regulator PGC1α, which in turn generates profound alterations in mitochondrial biogenesis, leading to a pathological accumulation of small mitochondria in mutant animals that contribute to cardiac disease. Notably, we demonstrate that systemic hypertension and HFpEF caused by miR-29 deficiency can be rescued by *PGC1α* haploinsufficiency, which reduces cardiac mitochondrial accumulation and extends longevity of miR-29–mutant mice. In addition, *PGC1α* is overexpressed in hearts from patients with HF. Collectively, our findings demonstrate the in vivo role of miR-29 in cardiovascular homeostasis and unveil a novel miR-29/PGC1α regulatory circuitry of functional relevance for cell metabolism under normal and pathological conditions.

## Author summary

To combat diseases, we first need to gain knowledge on how cells function at the molecular level to maintain normal physiology. One great scientific achievement of the last decade was the identification of thousands of small regulatory RNA molecules, called microRNAs. Strikingly, each microRNA has the potential to fine-tune the expression of hundreds of target genes depending on the spatiotemporal context. Therefore, defects in key microRNAs can contribute to the development of diseases. In the present work, we characterize the role for miR-29 in cardiac function in a mouse model. We found that mice deficient for miR-29 develop life-threatening cardiometabolic alterations that subsequently cause heart failure with diastolic dysfunction and systemic hypertension. We also demonstrate that these pathological phenotypes originate in part by the anomalous up-regulation of the transcriptional coactivator *PGC1α*, which can lead to mitochondrial hyperplasia in the heart. Genetic removal of one copy of *PGC1α* significantly attenuated the severity of the cardiovascular phenotype observed in miR-29–deficient mice. In addition, we show that *PGC1α* expression is misregulated in heart failure patients, suggesting that the implementation of miR-29 replacement therapy could potentially be used to treat these fatal pathologies.

## Introduction

Heart failure (HF) is a global epidemic affecting 1%–2% of the adult population (≥10% in the elderly) in developed countries [1]. HF is caused by the combined action of several genetic and environmental factors [2]. HF with preserved ejection fraction (HFpEF) is a highly prevalent form of HF, and it is defined as the inability of the heart to meet systemic demands despite normal ventricular contractile function. HFpEF is characterized by pulmonary edema, left ventricular (LV) diastolic impairment, and increased LV filling pressures. HFpEF is associated with comorbidities such as hypertension, atrial fibrillation, and diabetes [3]. The mechanisms leading to HFpEF are not fully understood and, consequently, there are no specific therapies for it.

Over the last decade, noncoding RNAs (ncRNAs) have emerged as essential regulators of organismal pathophysiology [4–6]. Recent studies have shown that different microRNAs (miRNAs), including those belonging to the miR-29 family, could play a role in the development of HF [7,8]. miR-29 is a family of miRNAs with three members (miR-29a, miR-29b, and miR-29c) encoded by two distinct genomic clusters: *miR-29a/b1* and *miR-29b2/c* [9]. These miRNAs play key roles in cancer [10], metabolism [11,12], apoptosis [13], fibrosis [14–16], neurological disorders [17–19], cardiovascular diseases [20–24], and aging [25,26]. To further elucidate the in vivo roles of miR-29 with special attention to cardiovascular disease, we have generated mice deficient in each miR-29 genomic cluster, as well as mice lacking both miR-29 loci.

In this work, we report that mice deficient in *miR-29a/b1* show reduced life span mainly because of the development of cardiometabolic alterations and HFpEF, which arise at least in part as a result of the altered expression of *PGC1α* [27]. Remarkably, these alterations in *miR-29a/b1*^*−/−*^ mice can be rescued or attenuated by *PGC1α* haploinsufficiency. Finally, we show that *PGC1α* is dysregulated in patients with HF. These results provide evidence for a novel regulatory pathway involving miR-29 and its bona fide target, PGC1α, which may have functional and clinical impact in cardiovascular diseases.

## Results

### Generation and phenotypic analysis of miR-29–deficient mice

To evaluate the in vivo roles of miR-29 family members, and considering their putative functional redundancy, we first generated mice deficient in each miR-29 genomic cluster. Embryonic stem (ES) cells heterozygous for a targeted deletion in *miR-29a/b1* and *miR-29b2/c* clusters, respectively, were produced following a homologous recombination strategy (S1a Fig). Heterozygosity was confirmed by Southern blot (S1b Fig). Afterwards, excision of the replacement cassette was performed using the Cre/loxP system. Mutant ES cells were used to generate chimeric mice that were then bred with C57BL/6N animals to generate heterozygous mice. After intercrossing these animals, we obtained *miR-29a/b1*^*−/−*^ and *miR-29b2/c*^*−/−*^ animals at a frequency consistent with the expected Mendelian ratio.

**Fig 1:**
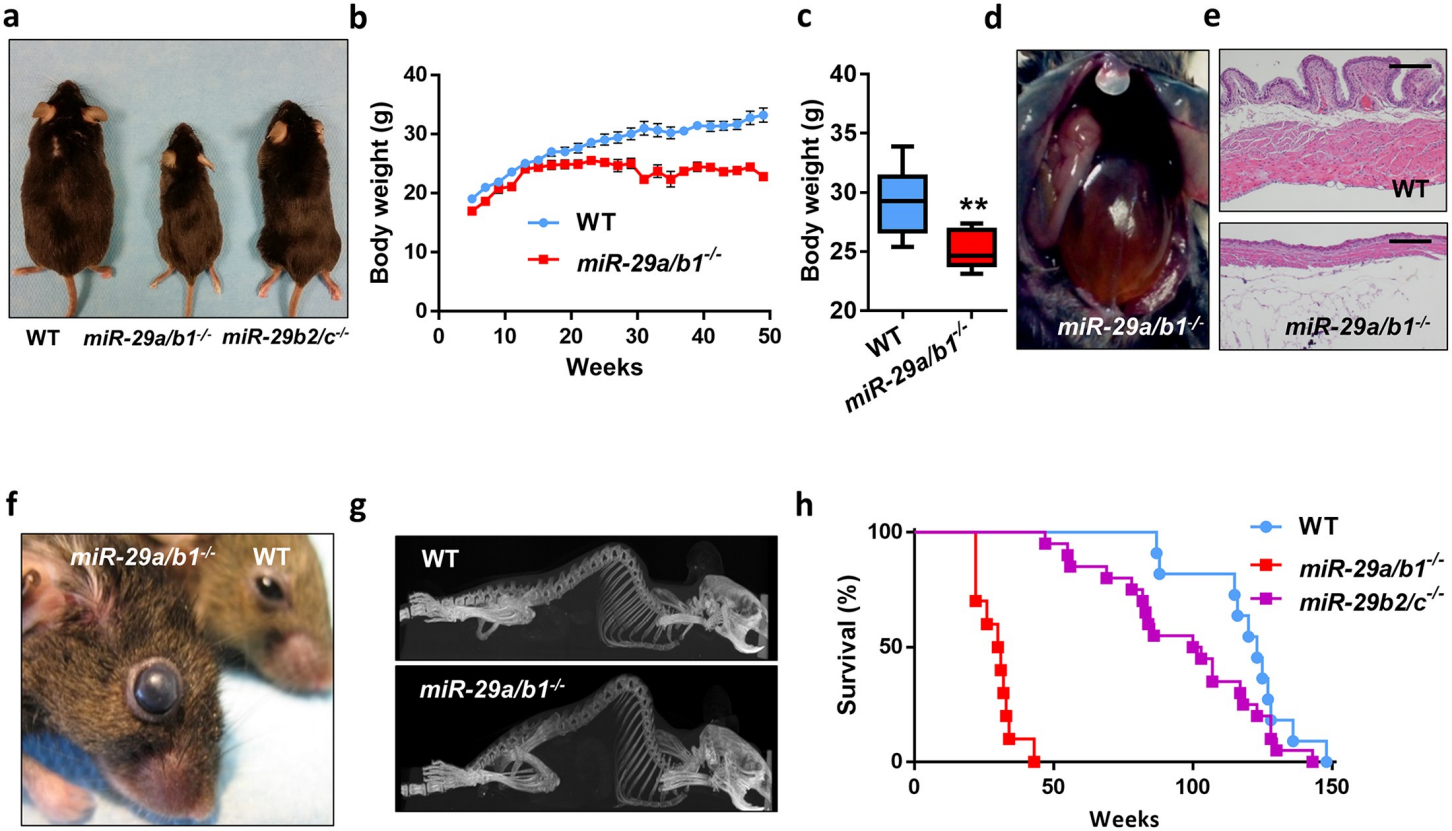
Phenotypic characterization of miR-29–deficient mice. (A) Representative picture of 8-month-old wild-type, *miR-29a/b1*^*−/−*^, *and miR-29b2/c*^*−/−*^ mice. (B) Body weight curves of wild-type (*n* = 9) and *miR-29a/b1*^*−/−*^ (*n* = 7) male mice (*p* < 0.05 from 25 to 49 weeks, two-tailed multiple Student *t* test, Bonferroni-corrected). (C) Body weight of 25-week-old wild-type (*n* = 9) and *miR-29a/b1*^*−/−*^ (*n* = 7) male mice. (D) Representative picture showing the urinary retention phenotype of 9-month-old *miR-29a/b1*^*−/−*^ mice. (E) HE sections of bladders of 7-month-old wild-type and *miR-29a/b1*^*−/−*^ mice (original magnification: ×4, scale bar: 200 μm). (F) Representative picture of the eyes of 9-month-old wild-type and *miR-29a/b1*^*−/−*^ mice. (G) CT scan of 7-month-old wild-type and *miR-29a/b1*^*−/−*^ female mice. (H) Kaplan-Meier survival plot of wild-type (*n* = 11), *miR-29a/b1*^*−/−*^ (*n* = 10), and *miR-29b2/c*^*−/−*^ (*n* = 20) mice (*p* < 0.0001 for the comparison between wild-type and *miR-29a/b1*^*−/−*^ mice; log-rank/Mantel-Cox test). Original raw data can be found in S1 Data file. CT, computed tomography; HE, hematoxylin–eosin; WT, wild-type.

Mice lacking the *miR-29a/b1* cluster were morphologically indistinguishable from their wild-type littermates until the fourth month of life. Subsequently, mutant mice developed a fully penetrant phenotype characterized by a significant growth retardation and body weight reduction (Fig 1a–1c), which can be partially explained by a dramatic reduction in white and brown adipose tissue (S2a and S2b Fig). Moreover, *miR-29a/b1*–deficient mice exhibited smaller adipocytes, compared with wild-type animals (S2c–S2e Fig). Nevertheless, the most apparent alterations in these mice were their remarkable urinary retention and severe bladder distension (Fig 1d). The growing pressure inside the bladder was accompanied by urothelium and muscular layer atrophy (Fig 1e). *miR-29a/b1*^*−/−*^ mice also showed increased intraocular pressure, which leads to eye degeneration and blindness (Fig 1f). Additionally, these mice were sterile and exhibited an abnormal posture characterized by a hunched position and the development of thoracic kyphosis and severe ataxia (Fig 1g and S1 Video). As a consequence of all these phenotypic alterations, *miR-29a/b1–*mutant mice suffered premature death, with a median life span of 30 weeks (control littermates, 123 weeks, *p* < 0.0001) (Fig 1h). In marked contrast, *miR-29b2/c*^*−/−*^ mice developed normally without any obvious alterations (Fig 1a), except a slight decrease in body weight. (S3 Fig). Interestingly, old *miR-29b2/c*^*−/−*^ mice developed urinary retention in a similar manner to that observed in *miR-29a/b1*^*−/−*^ mice, although the penetrance of this phenotypic alteration was incomplete.

We found very remarkable that the phenotypic alterations in both miR-29 mutant models were dramatically different. A possible explanation for this phenomenon could be that the members of this miRNA family display different target specificities. However, we ruled out this hypothesis, because all three members share exactly the same seed sequence, and in line with this, previous experiments from us and others showed a similar level of repression on target genes for the three miR-29 members in luciferase experiments [25,28]. Another explanatory hypothesis could be that the two clusters show differential tissue expression. To evaluate this, we took advantage of publicly available Encyclopedia of DNA Elements (ENCODE) microRNA-Seq data from different mouse tissues to study the expression of the miR-29 family (S4a Fig). This analysis revealed that (1) the three members of the miR-29 family are ubiquitously expressed across the analyzed mouse tissues; (2) miR-29a is the dominant member in most tissues studied, accounting for more than 50% of total miR-29 levels in all tissues; (3) miR-29b is the less abundantly expressed member, and its expression correlates highly with miR-29a but not with miR-29c (S4b Fig); (4) miR-29c expression seems to be more important in neural tissues. In summary, these results indicate that the *miR-29a/b1* cluster exerts a stronger functional role, being the main source of miR-29, and therefore it is not surprising that *miR-29b2/c*–null mice show a much milder phenotype compared with *miR-29a/b1*–deficient mice. We also confirmed this hypothesis by measuring miR-29 expression patterns in heart, kidney, and liver from our *miR-29a/b1*^*−/−*^ and *miR-29b2/c*^*−/−*^ mouse models using quantitative reverse transcription PCR (RT-qPCR) (S5a Fig).

**Fig 2:**
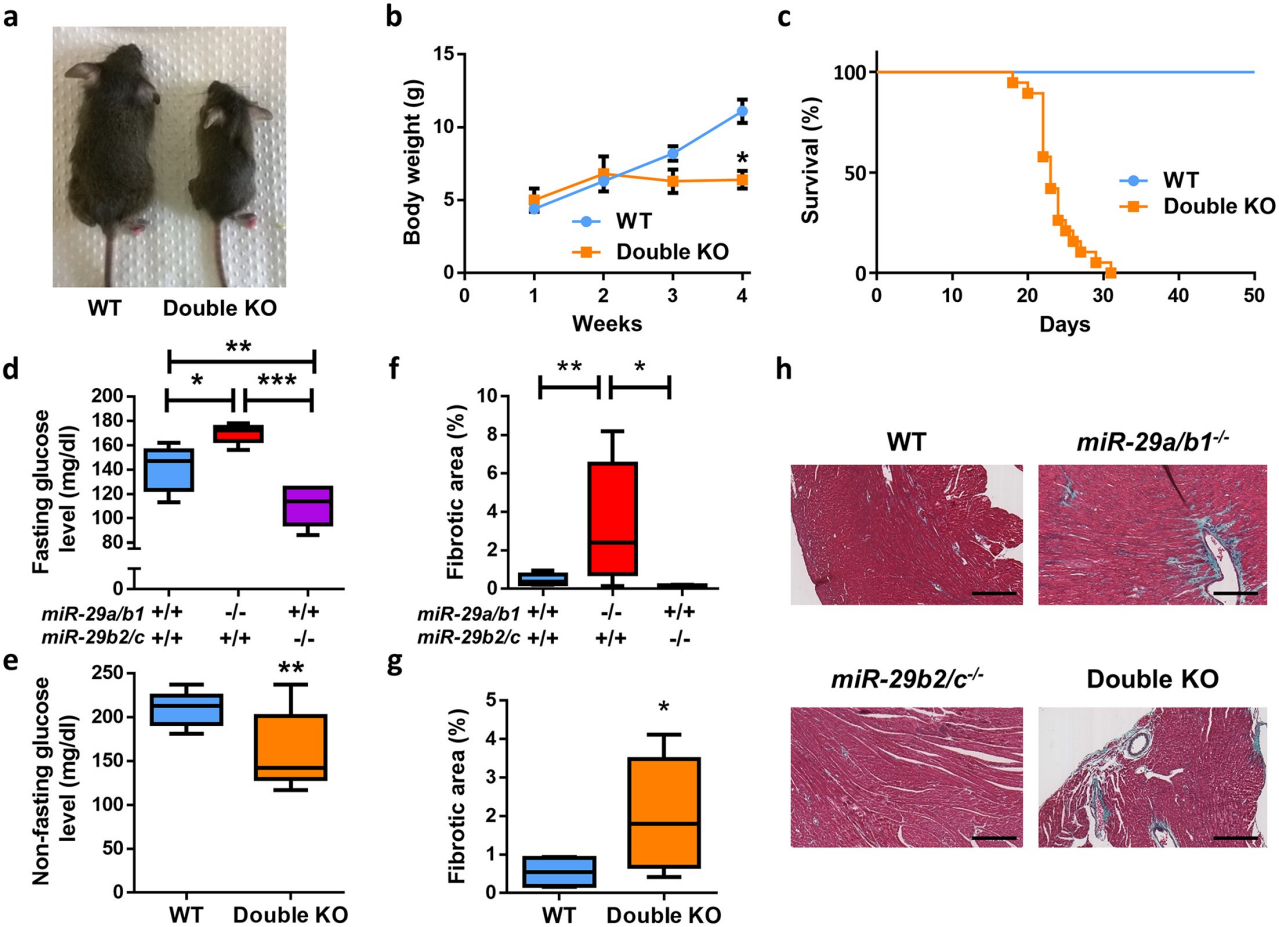
Cardiometabolic alterations of miR-29–deficient mice. (A) Representative picture of 22-day-old wild-type and double KO mice. (B) Body weight curves of wild-type (*n* = 15) and double KO (*n* = 3) mice (*p* < 0.05 at 4 weeks, two-tailed multiple Student *t* test, Bonferroni-corrected). (C) Kaplan–Meier survival plot of wild-type (*n* = 11) and double KO (*n* = 19) mice (*p* < 0.0001; log-rank/Mantel-Cox test). (D) Fasting blood glucose level in wild-type (*n* = 10), *miR-29a/b1*^*−/−*^ (*n* = 5), and *miR-29b2/c*^*−/−*^ (*n* = 6) mice. (E) Nonfasting blood glucose levels in wild-type (*n* = 9) and double KO (*n* = 12) mice (two-tailed Student *t* test with Welch’s correction). (F) Percentage of collagen present in cardiac sections of wild-type (*n* = 12), *miR-29a/b1*^*−/−*^ (*n* = 8), and *miR-29b2/c*^*−/−*^ (*n* = 5) mice. (G) Percentage of collagen present in cardiac sections of wild-type (*n* = 4) and double KO (*n* = 10) mice (two-tailed Student *t* test with Welch’s correction). (H) Gomori’s trichrome stained heart sections of wild-type, *miR-29a/b1*^*−/−*^, *miR-29b2/c*^*−/−*^, and double KO mice (original magnification: ×10, scale bar: 200 μm). Original raw data can be found in S1 Data file. KO, knock-out; WT, wild-type.

The generation of *miR-29a/b1*^*−/−*^ *miR-29b2/c*^*−/−*^ double knock-out (KO) mice, lacking both miR-29 clusters, was carried out by intercrossing *miR-29a/b1*^*+/−*^ and *miR-29b2/c*^*−/−*^ mice. The absence of miR-29 expression in double KO mice was verified by RT-qPCR in heart, lung, and kidney (S5b Fig). Double KO mice exhibited more pronounced alterations than single KOs, with their striking growth retardation and reduced body weight few days after birth being especially remarkable (Fig 2a and 2b). In addition, the life span of mutant mice deficient in both miR-29 clusters was dramatically reduced to a median of 23 days (*p* < 0.0001) (Fig 2c).

### Cardiometabolic alterations in miR-29–deficient mice

Aimed at elucidating the underlying causes of premature death of miR-29–deficient mice, we first focused on putative metabolic abnormalities [11,29,30]. These analyses revealed that fasted *miR-29a/b1*^*−/−*^ mice exhibit hyperglycemia (Fig 2d), probably because of their reduced serum insulin concentration and the up-regulation of the gluconeogenic pathway in the liver (S6 and S7a Figs), two features frequently present in diabetes mellitus patients [31,32]. Additionally, and consistent with the lack of apparent phenotypic alterations, we did not observe any significant changes in key hepatic metabolic pathways in *miR-29b2/c*^*−/−*^ mice. Nevertheless, both serum glucose and insulin concentrations were reduced in these animals (Fig 2d and S6 and S7a Figs). Nonfasted double KO mice showed a significant reduction in their glycemia in comparison with their wild-type littermates (Fig 2e). Obviously, the nonfasting status of double KO animals, given the impossibility of fasting infant mice, may affect the outcome of glucose measurements. However, the up-regulation of key metabolic enzymes of the glycolytic pathway in these double KO mice could also explain this paradoxical result (S7b Fig).

Next, we examined the potential antifibrotic role of miR-29 previously suggested by different in vitro and in vivo studies [14,15]. We found necrotic areas with collagen accumulation in mutant hearts from *miR-29a/b1*^*−/−*^, but not in those from *miR-29b2/c*^*−/−*^ (Fig 2f–2h). This accumulation of fibrotic collagenous material was also evident and abundant in double KO mice. These findings prompted us to speculate that double KO mice could die prematurely because of cardiometabolic alterations. Given that the life span of these mice is reduced to less than 4 weeks, making it impossible to perform a deep cardiovascular characterization of these animals, we focused on *miR-29a/b1*^*−/−*^ mice in order to characterize the biological roles of the miR-29 family in cardiovascular physiopathology.

### *miR-29a/b1*^*−/−*^ mice develop HFpEF

**Fig 3:**
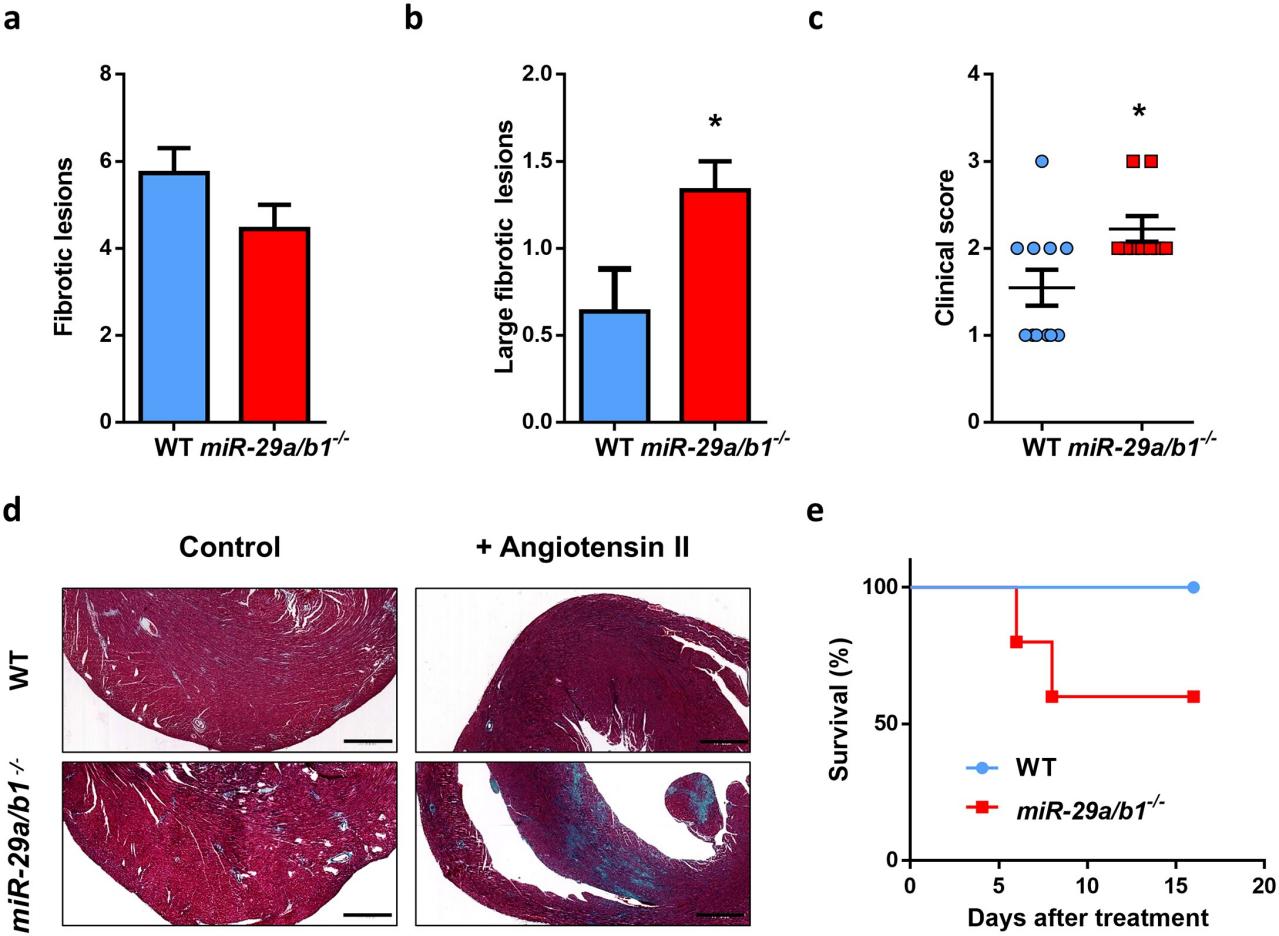
*miR-29a/b1*^*−/−*^ mice display increased susceptibility to angiotensin II–induced cardiac fibrosis. (A) Median of fibrotic lesions, (B) large fibrotic lesions, and (C) clinical score (grade 0: no fibrotic areas; grade 1: less than 25%; grade 2: from 26% to 50%; grade 3: from 51% to 75%; and grade 4: more than 76% of myocardium affected) in 3-month-old wild-type (*n* = 11) and *miR-29a/b1*^*−/−*^ (*n* = 9) mice. (D) Representative micrographs of wild-type and *miR-29a/b1*^*−/−*^ mice treated with angiotensin-II for 6 days (Gomori’s trichrome staining, original magnification: ×4, scale bar: 500 μm). (E) Kaplan–Meier survival plot of wild-type (*n* = 5) and *miR-29a/b1*^*−/−*^ (*n* = 5) mice treated with angiotensin-II for 6 days. Original raw data can be found in S1 Data file. WT, wild-type.

To further deepen the abovementioned observations, we sought to evaluate if the fibrotic pathway was exacerbated in the hearts with *miR-29a/b1*–deficient background. For this purpose, we induced cardiac fibrosis by the administration of angiotensin-II through osmotic pumps to 3-month-old *miR-29a/b1*^*−/−*^ (free of any pathological alterations) and wild-type mice. Even though the number of fibrotic lesions per mouse was similar in both genotypes, *miR-29a/b1*^*−/−*^ mice showed a 3-fold increment in large lesions (>400 μm) and a worse clinical score in comparison with age-matched controls (Fig 3a–3d). Moreover, *miR-29a/b1*^*−/−*^ mice exhibited a higher susceptibility to angiotensin-II–induced cardiac HF, as 40% of mutant mice died during the treatment (Fig 3e).

**Fig 4:**
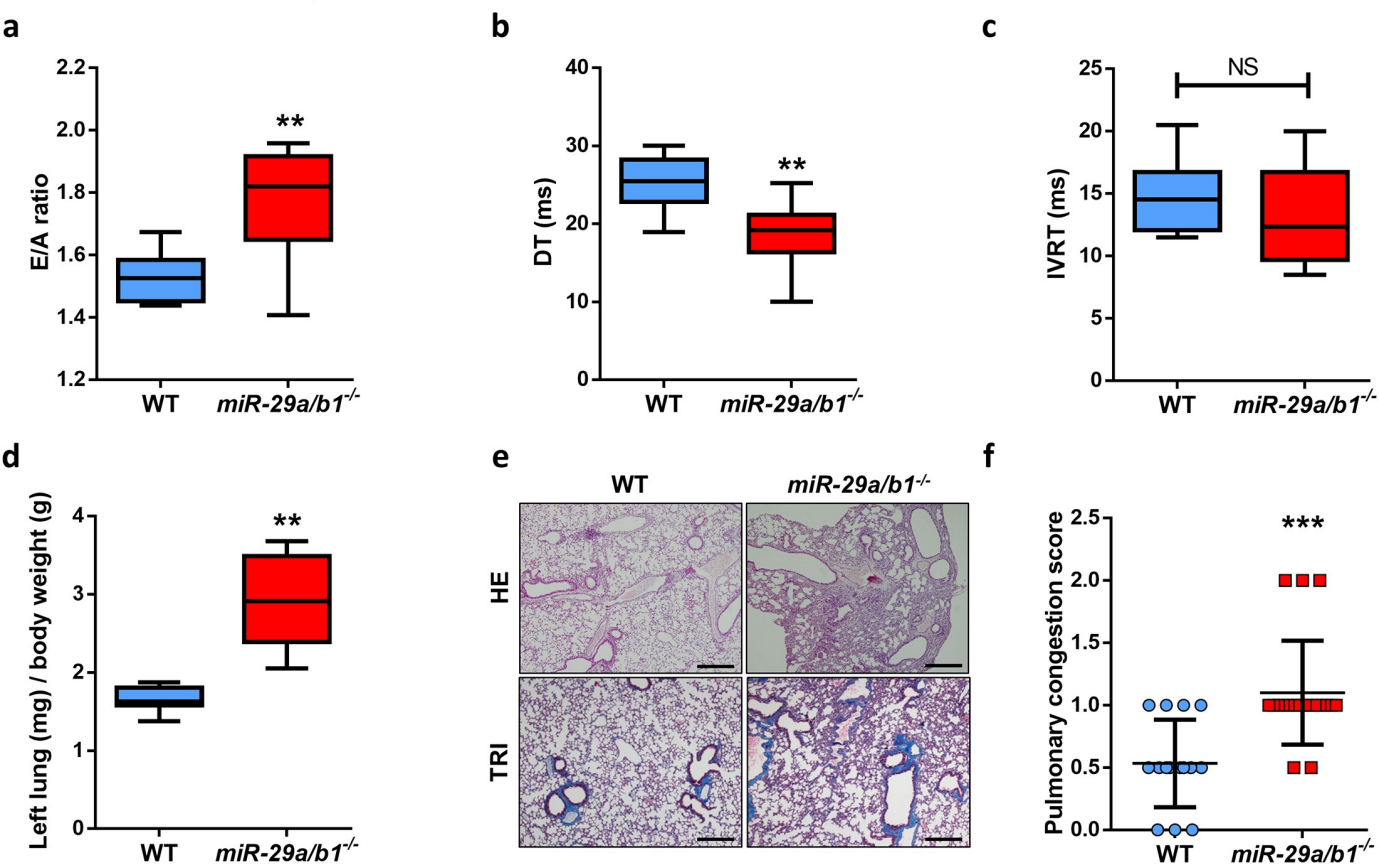
*miR-29a/b1*^*−/−*^ mice develop HFpEF. (A) Quantification of E/A fraction in wild-type (*n* = 10) and *miR-29a/b1*^*−/−*^ (*n* = 9) mice (two-tailed Student *t* test with Welch’s correction). (B) Quantification of DT of early filling in wild-type (*n* = 10) and *miR-29a/b1*^*−/−*^ (*n* = 9) mice. (C) Quantification of IVRT in wild-type (*n* = 10) and *miR-29a/b1*^*−/−*^ (*n* = 9) mice. (D) Ratio of left lung weight to body weight in wild-type (*n* = 8) and *miR-29a/b1*^*−/−*^ (*n* = 5) mice (two-tailed Student *t* test with Welch’s correction). (E) HE and Masson’s trichrome stained lung sections of wild-type and *miR-29a/b1*^*−/−*^ mice (original magnification: ×4, scale bar: 500 μm). (F) Clinical score of pulmonary congestion in wild-type (*n* = 15) and *miR-29a/b1*^*−/−*^ (*n* = 20) mice. Original raw data can be found in S1 Data file. DT, deceleration time; E/A, early and late diastolic filling velocities ratio; HE, hematoxylin–eosin; HFpEF, heart failure with preserved ejection fraction; IVRT, isovolumetric relaxation time; NS, non-significant; TRI, Masson’s trichrome; WT, wild-type.

To assess the in vivo relevance of cardiac alterations in *miR-29a/b1*^*−/−*^ mice, we performed a more comprehensive study of the cardiovascular system in these mutant animals. Two-dimensional echocardiographic studies revealed that LV wall thickness, LV end-diastolic and end-systolic diameters, and LV ejection fraction were similar in wild-type and age-matched *miR-29a/b1*^*−/−*^ mice (S8a–S8d Fig). Conversely, *miR-29a/b1*^*−/−*^ showed overt LV diastolic dysfunction with a restrictive filling pattern, evidenced by an abnormal early (E) and late (A) diastolic filling velocities ratio (E/A), and a shortening in the deceleration time (DT) of early filling compared with wild-type animals (Fig 4a and 4b). There is also a slight decrease in the isovolumetric relaxation time (IVRT) in mutant animals (Fig 4c). As a consequence, lungs from *miR-29a/b1*^*−/−*^ mice were severely congestive in relation to wild-type mice, evidenced by the increase in relative lung weight, the occurrence of alveolar edema (Fig 4d and 4e), and, consequently, a worse pulmonary congestion score (Fig 4f). Taken together, these features (fibrotic LV, diastolic dysfunction, and congestive lungs) resemble a HFpEF syndrome.

At the molecular level, we measured the cardiac expression of key metabolic enzymes of the β-oxidation and glycolytic pathways, as well as *Glut1*, a glucose transporter (S9 Fig). We found a significant reduction in the expression of β-oxidation genes, *Acadvl*, *Cpt1a*, and *Cpt1b*, while, conversely, *Glut1* was up-regulated in mutant animals. The expression of glycolytic genes did not change. These results suggest that there is a metabolic switch in fuel utilization, from fatty acids to glucose, in the hearts of *miR-29a/b1*^*−/−*^ mice. This switch has been previously reported in other mouse HF models [33].

### *miR-29a/b1*^*−/−*^ mice develop systemic hypertension

**Fig 5:**
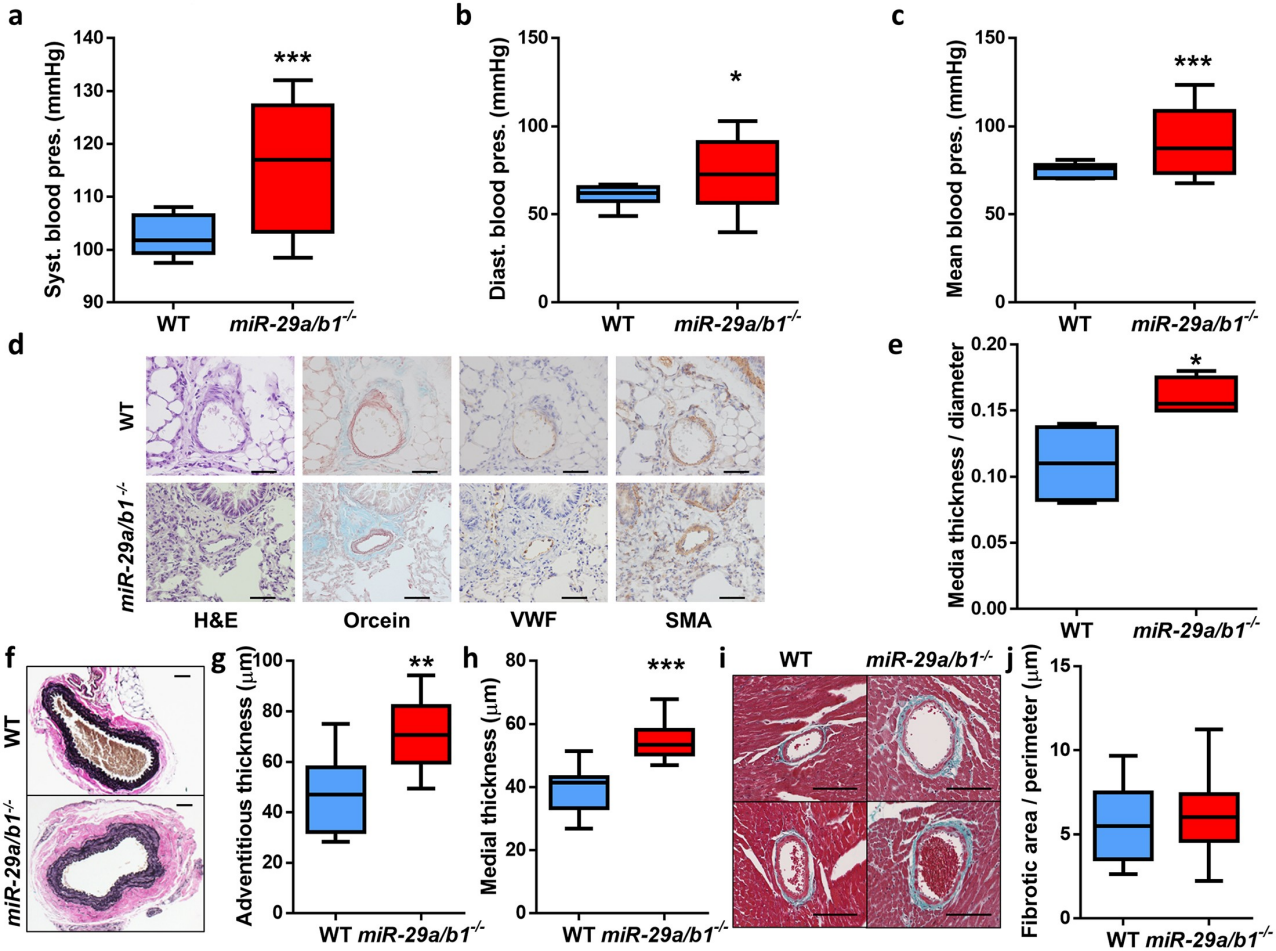
Systemic hypertension and vascular remodeling in *miR-29a/b1*^*−/−*^ mice. (A) Systolic, (B) diastolic, and (C) mean blood pressure values from wild-type (*n* = 10) and *miR-29a/b1*^*−/−*^ (*n* = 8) mice. (D) Hematoxylin–eosin (HE), orcein for elastic fibers (Orcein), von Willebrand factor (VWF), and α-smooth muscle actin (SMA) staining of small pulmonary lung vessels (<50 μm) of wild-type and *miR-29a/b1*^*−/−*^ mice (original magnification: ×40, scale bars: 50 μm). (E) Quantification of media layer thickness/diameter ratio of five SMA-stained small pulmonary blood vessels per mouse in wild-type (*n* = 4) and *miR-29a/b1*^*−/−*^ (*n* = 4) mice. (F) Micrographs of Verhoeff–Van Gieson elastic staining of aortas from representative wild-type and *miR-29a/b1*^*−/−*^ mice (original magnification: ×10, scale bar: 50 μm). (G) Thickness of the aortic adventitia layer of wild-type (*n* = 10) and *miR-29a/b1*^*−/−*^ mice (*n* = 11). (H) Thickness of the aortic media layer of wild-type (*n* = 9) and *miR-29a/b1*^*−/−*^ (*n* = 11) mice. (I) Representative micrographs of coronary arteries stained with Masson’s trichrome of wild-type and *miR-29a/b1*^*−/−*^ mice (original magnification: ×20, scale bars: 100 μm). (J) Quantification of the surrounding fibrotic area/perimeter ratio of six coronary arteries per mouse in wild-type (*n* = 4) and *miR-29a/b1*^*−/−*^ (*n* = 9) mice. Original raw data can be found in S1 Data file. Diast., diastolic; HE, hematoxylin–eosin; Orcein, orcein for elastic fibers; pres., pressure; SMA, α-smooth muscle actin; Syst., systolic; VWF, von Willebrand factor; WT, wild-type.

Subsequently, we looked for pathological alterations in the vascular system and found that *miR-29a/b1*^*−/−*^ mice presented higher levels of systemic blood pressure compared with wild-type animals (Fig 5a–5c). Because vascular remodeling is a concomitant phenomenon of impaired cardiac function and hypertension, we analyzed small pulmonary blood vessels. We found that *miR-29a/b1*^*−/−*^ mice developed hypertrophy of small pulmonary arteries (Fig 5d) with thickening of the tunica media (Fig 5e). Then, we searched for alterations in other blood vessels and found aortic abnormalities consisting of fibrotic accumulation in the tunica adventitia and medial thickening in the vessel wall of mutant mice. Verhoeff–Van Gieson staining of aortas confirmed the higher amount of elastic fibers in mutant animals (Fig 5f–5h). The aortic fibrosis in *miR-29a/b1*^*−/−*^ mice is suggestive of increased vascular stiffness. Conversely, we found no changes in fibrotic accumulation in the coronary arteries of *miR-29a/b1*^*−/−*^ mice compared with wild-type animals (Fig 5i and 5j).

Given the dramatic urinary retention in mutant animals, and in order to elucidate if renal failure is responsible for the increase in blood pressure, we studied the urinary system in *miR-29a/b1*^*−/−*^ mice. Serum creatinine level was lower in *miR-29a/b1*^*−/−*^ than in wild-type mice (S10a Fig). However, histological analysis of kidneys did not reveal any morphological alteration (S10b Fig).

Taken collectively, the occurrence of metabolic disorders, cardiac fibrosis, diastolic dysfunction, and pulmonary congestion, together with systemic hypertension and vascular remodeling, suggests that *miR-29a/b1*^*−/−*^ mice develop HFpEF because of a common cardiometabolic deregulated mechanism that leads to premature death.

### *PGC1α* haplodeficiency rescues cardiac function in *miR-29a/b1*^*−/−*^ mice

To gain mechanistic insights into the cardiometabolic phenotypes found in mice deficient in miR-29, we performed a candidate analysis approach to identify miR-29 targets whose deregulated expression could contribute to the observed alterations in miR-29–mutant mice. For this purpose, we carried out full transcriptome analysis in hearts from miR*-29a/b1*^*−/−*^ and miR*-29b2/c*^*−/−*^ mice and focused on up-regulated genes with predicted or validated miR-29 binding sites. Consistent with the less relevant role of *miR-29b2/c* in the heart, mice deficient in this cluster showed a reduced number of differentially expressed genes (373) compared with *miR-29a/b1*^*−/−*^ mice (1842) (S11a Fig and S1 Table). Importantly, only six miR-29 predicted target genes were found up-regulated in *miR-29b2/c*^*−/−*^ mice, none of them differentially expressed in *miR-29a/b1*^*−/−*^ mice (S11b and S11c Fig). In contrast, 56 genes with predicted miR-29 binding sites were found up-regulated in hearts from *miR-29a/b1*^*−/−*^ mice (S11b and S11c Fig), including experimentally validated targets such as *Hdac4*, *Klf4*, *Foxo3*, *Per1*, *Bmf*, *Mmp2*, *Lox*, and several collagens, among others [13,34–40].

We found particularly interesting that elastin (*Eln*) was significantly up-regulated in *miR-29a/b1*^*−/−*^ mice, because the protein encoded by this gene is a major component of the extracellular matrix. Additionally, *Eln* has been experimentally validated as a miR-29 target and plays important roles in the cardiovascular system [41,42]. Furthermore, we have showed increased accumulation of elastic fibers in aortas from *miR-29a/b1*–deficient mice (Fig 5f), and *Eln* overexpression in mutant mice was confirmed by RT-qPCR (S12a Fig). Consequently, to test whether *Eln* up-regulation might be involved in the phenotypic changes of mutant mice, we attempted to rescue the phenotypic alterations of *miR-29a/b1*–null mice by generating *miR-29a/b1*^*−/−*^ animals haploinsufficient for *Eln* gene (S12b Fig). However, this intervention did not improve the life span of *miR-29a/b1*^*−/−*^ mice, suggesting that *Eln* up-regulation does not play a critical role in the development of the phenotypic alterations present in these mice.

Remarkably, we found *PGC1α* among the top 10 up-regulated miR-29 target genes in *miR-29a/b1*^*−/−*^ mice (S11c Fig). *PGC1α*, a predicted target of all members of the miR-29 family (S13 Fig), drives many metabolic pathways in the cardiovascular system, and its cardiac-specific overexpression results in mitochondrial alterations in cardiomyocytes and in the development of cardiomyopathy [43–46]. Furthermore, *PGC1α* has been experimentally validated as a bona fide miR-29 target in mouse liver, where it controls hepatic glucose production and glucose tolerance [30]. Therefore, we hypothesized that the *PGC1α* up-regulation caused by miR-29 deficiency may trigger an exacerbated mitochondrial biogenesis and contribute, together with other alterations, to the development of LV diastolic dysfunction with HFpEF and the other cardiovascular abnormalities observed in *miR-29a/b1*^*−/−*^ mice.

**Fig 6:**
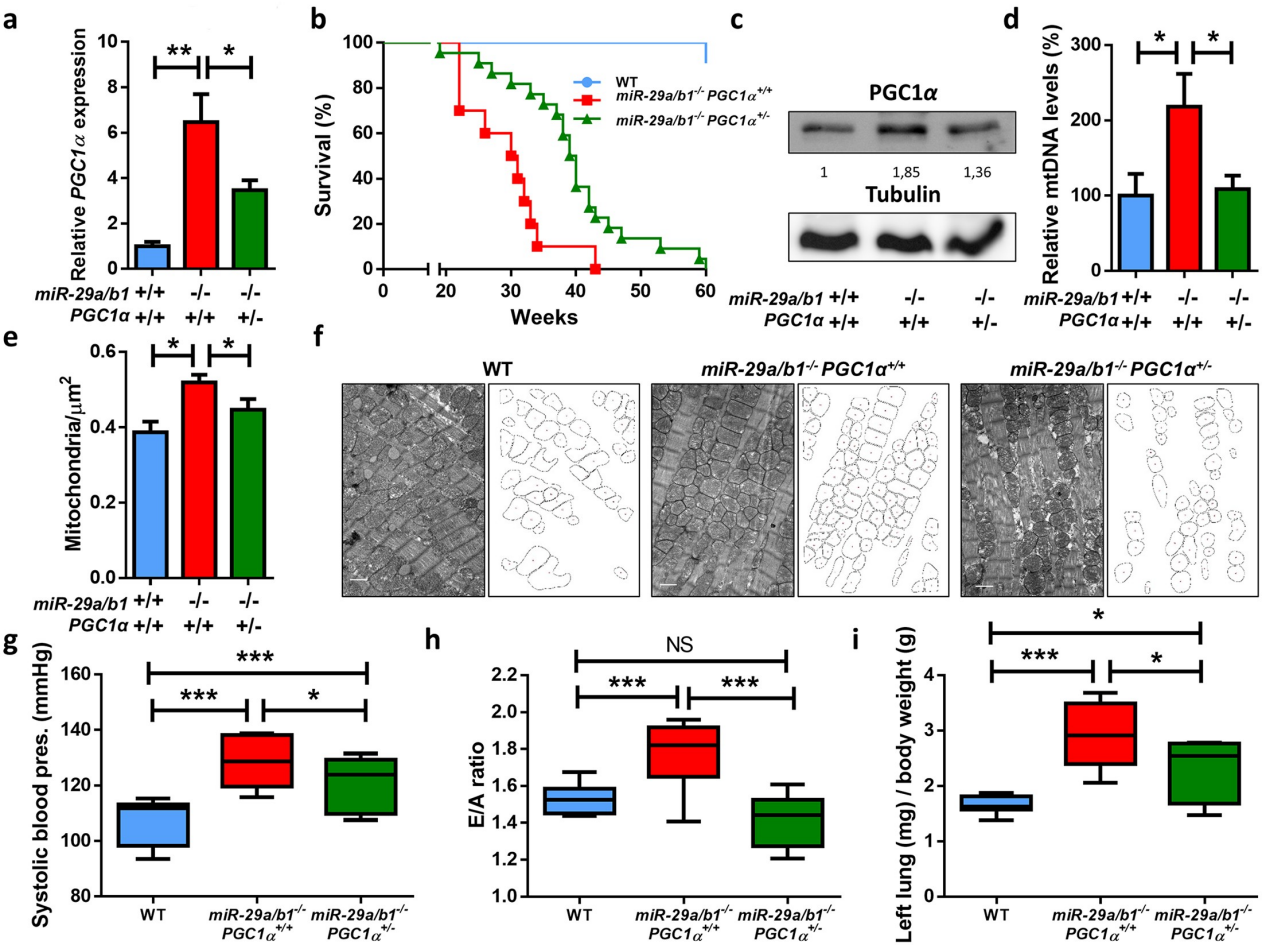
*PGC1α* is up-regulated in *miR-29a/b1*^*−/−*^ mice, and its reduction extends life span and rescues cardiovascular pathology. (A) *PGC1α* expression in hearts from wild-type (*n* = 7), *miR-29a/b1*^*−/−*^ *PGC1α*^*+/+*^ (*n* = 13), and *miR-29a/b1*^*−/−*^ *PGC1α*^*+/−*^ (*n* = 7) mice. (B) Kaplan–Meier survival plot of wild-type (*n* = 11), *miR-29a/b1*^*−/−*^ *PGC1α*^*+/+*^ (*n* = 10), and *miR-29a/b1*^*−/−*^ *PGC1α*^*+/−*^ (*n* = 22) mice (*p* < 0.01 for the comparison between *miR-29a/b1*^*−/−*^ *PGC1α*^*+/+*^ and *miR-29a/b1*^*−/−*^ *PGC1α*^*+/−*^ mice; log-rank/Mantel-Cox test Bonferroni-corrected for multiple comparisons). (C) Western blot of PGC1*α* in hearts from wild-type, *miR-29a/b1*^*−/−*^ *PGC1α*^*+/+*^, and *miR-29a/b1*^*−/−*^ *PGC1α*^*+/−*^ mice (relative densitometric quantification represented as arbitrary units). (D) Analysis of mtDNA quantity expressed as a percentage of levels in wild-type (*n* = 6), *miR-29a/b1*^*−/−*^ *PGC1α*^*+/+*^ (*n* = 4), and *miR-29a/b1*^*−/−*^ *PGC1α*^*+/−*^ (*n* = 6) mice. (E) Number of mitochondria per μm^2^ in wild-type (six micrographs from two different mice), *miR-29a/b1*^*−/−*^ *PGC1α*^*+/+*^ (22 micrographs from four different mice), and *miR-29a/b1*^*−/−*^ *PGC1α*^*+/−*^ (22 micrographs from three different mice) animals. (F) Electron micrographs and schematic representation of mitochondria of hearts from wild-type, *miR-29a/b1*^*−/−*^ PGC1α^+/+^, and *miR-29a/b1*^*−/−*^ *PGC1α*^*+/−*^ mice (original magnification: ×15.000, scale bar: 1 μm). (G) Systolic blood pressure values from wild-type (*n* = 10), *miR-29a/b1*^*−/−*^ *PGC1α*^*+/+*^ (*n* = 8), and *miR-29a/b1*^*−/−*^ *PGC1α*^*+/−*^ (*n* = 6) mice. (H) Quantification of E/A fraction in wild-type (*n* = 10), *miR-29a/b1*^*−/−*^ *PGC1α*^*+/+*^ (*n* = 9), and *miR-29a/b1*^*−/−*^ *PGC1α*^*+/−*^ (*n* = 6) mice. (I) Ratio of left lung weight to body weight in wild-type (*n* = 8), *miR-29a/b1*^*−/−*^ *PGC1α*^*+/+*^ (*n* = 5), and *miR-29a/b1*^*−/−*^ *PGC1α*^*+/−*^ (*n* = 5) mice. Original raw data can be found in S1 Data file. E/A, early and late diastolic filling velocities ratio; mtDNA, mitochondrial DNA; NS, non-significant; WT, wild-type.

To evaluate this hypothesis, we first validated cardiac overexpression of *PGC1α* by RT-qPCR in *miR-29a/b1*^*−/−*^ animals and observed a 6-fold increase in these mutant mice compared with their wild-type littermates (Fig 6a). Moreover, *PGC1α* was also up-regulated in the liver and kidney of *miR-29a/b1*^*−/−*^ mice (S14a Fig). However, and consistent with the lack of cardiovascular disease, *PGC1α* was unaltered in *miR-29b2/c*^*−/−*^ mice (S14b Fig). Importantly, analysis of ENCODE transcriptome data from mouse tissues with matched microRNA-Seq data revealed that *PGC1α* shows its highest level of expression in the heart, and there is a remarkable negative correlation between *PGC1α* expression and total levels of miR-29 across the analyzed tissues (S15a and S15b Fig). These results reinforce the existence of a PGC1α/miR-29 regulatory axis of special impact in heart tissue.

Given that miRNAs often control molecular pathways by targeting multiple components, we inspected the gene expression changes in miR-29–deficient mice to find *PGC1α*-interacting genes with deregulated expression. Notably, we detected significant up-regulation in hearts from *miR-29a/b1*^*−/−*^ mice of genes encoding PGC1α-activated proteins *Pparα* and *Pparδ*, the latter being also a predicted miR-29 target gene, according to TargetScan (S1 Table). However, we did not observe significant changes in *Pparγ* gene expression, a result that was confirmed by western blot (S16 Fig). *Rora*, a predicted miR-29 target gene encoding another *PGC1α* coactivated protein, was also found up-regulated in miR-29–deficient mice. Furthermore, gene set enrichment analysis using the Kyoto Encyclopedia of Genes and Genomes (KEGG) database yielded numerous significantly deregulated pathways in *miR-29a/b1*–deficient mice (S17a Fig). Remarkably, metabolic pathways linked to *PGC1α* signaling, such as the citrate cycle, oxidative phosphorylation, or glycolysis/gluconeogenesis, were found enriched in this analysis (S17b Fig). Differentially expressed genes in hearts from *miR-29a/b1*^*−/−*^ mice were also enriched in multiple important signaling networks, such as the energy-sensing AMP-activated protein kinase (AMPK) pathway or the stress response p53 signaling network, among others. Importantly, highly relevant pathways for heart function, such as the regulation of cytoskeleton, cardiac muscle contraction, gap junctions, and cyclic AMP (cAMP) and calcium signaling, were also significantly enriched in differentially expressed genes from *miR-29a/b1*–deficient mice. Moreover, consistently with the lack of phenotype in *miR-29c/b2*^*−/−*^ mice, neither the *PGC1α*-interacting proteins nor the vast majority of the enriched pathways were affected in hearts from these mice (S17b Fig).

Next, we intercrossed miR-29–mutant animals with mice heterozygous for *PGC1α* and found that *miR-29a/b1*^*−/−*^ *PGC1α*^*+/−*^ mice had a significant life span extension compared with *miR-29a/b1*^*−/−*^ *PGC1α*^*+/+*^ mice (39.5 versus 30 weeks, *p* < 0.01) (Fig 6b). Notably, the haploinsufficiency of *PGC1α* significantly reduced the expression levels of this gene in *miR-29a/b1*^*−/−*^ *PGC1α*^*+/−*^ mice (Fig 6a). Furthermore, western blot analysis corroborated the increase in PGC1α protein levels in miR-29–mutant mice and its reduction in *miR-29a/b1*^*−/−*^ *PGC1α*^*+/−*^ animals (Fig 6c).

Given the central role of PGC1α in mitochondrial biology [47], we analyzed the putative occurrence of mitochondrial alterations in the heart from both *miR-29a/b1*^*−/−*^ *PGC1α*^+/+^ and *miR-29a/b1*^*−/−*^ *PGC1α*^*+/−*^ mice. We first examined heart mitochondrial DNA (mtDNA) levels, a good indicator of mitochondrial number. Notably, we found a significant increase in cardiac mtDNA levels in *miR-29a/b*^*−/−*^ PGC1*α*^+/+^ mice compared with age-matched controls, which were restored to wild-type levels in *miR-29a/b1*^*−/−*^ *PGC1α*^*+/−*^ mice (Fig 6d). Transmission electron microscopy (TEM) studies confirmed an increased number of mitochondria per μm^2^ in *miR-29a/b1*^*−/−*^ *PGC1α*^*+/+*^, which was recovered in *miR-29a/b1*^*−/−*^ *PGC1α*^*+/−*^ hearts (Fig 6e). We also observed that mitochondrial size was significantly smaller in both mutant models (S18 Fig). Electron micrographs showed the accumulation of smaller rounded mitochondria in *miR-29a/b1*^*−/−*^ *PGC1α*^*+/+*^ cardiomyocytes and the rescue of this phenotype in *miR-29a/b1*^*−/−*^ *PGC1α*^*+/−*^ animals (Fig 6f). Importantly, *miR-29a/b*^*−/−*^ *PGC1α*^*+/−*^ mice showed a significant reduction in systemic blood pressure compared with *miR-29a/b1*^*−/−*^ *PGC1α*^*+/+*^ mice (Fig 6g and S19a Fig). Furthermore, several analyzed markers of diastolic dysfunction were also reverted in *miR-29a/b1*^*−/−*^ *PGC1α*^*+/−*^ mice (Fig 6h and 6i and S19b and S19c Fig).

Collectively, these results demonstrate the occurrence of mitochondrial abnormalities in cardiac tissue from *miR-29a/b1*^*−/−*^ mice and the extensive rescue of these alterations by *PGC1α* haploinsufficiency.

### *PGC1α* expression is deregulated in patients with HF

**Fig 7:**
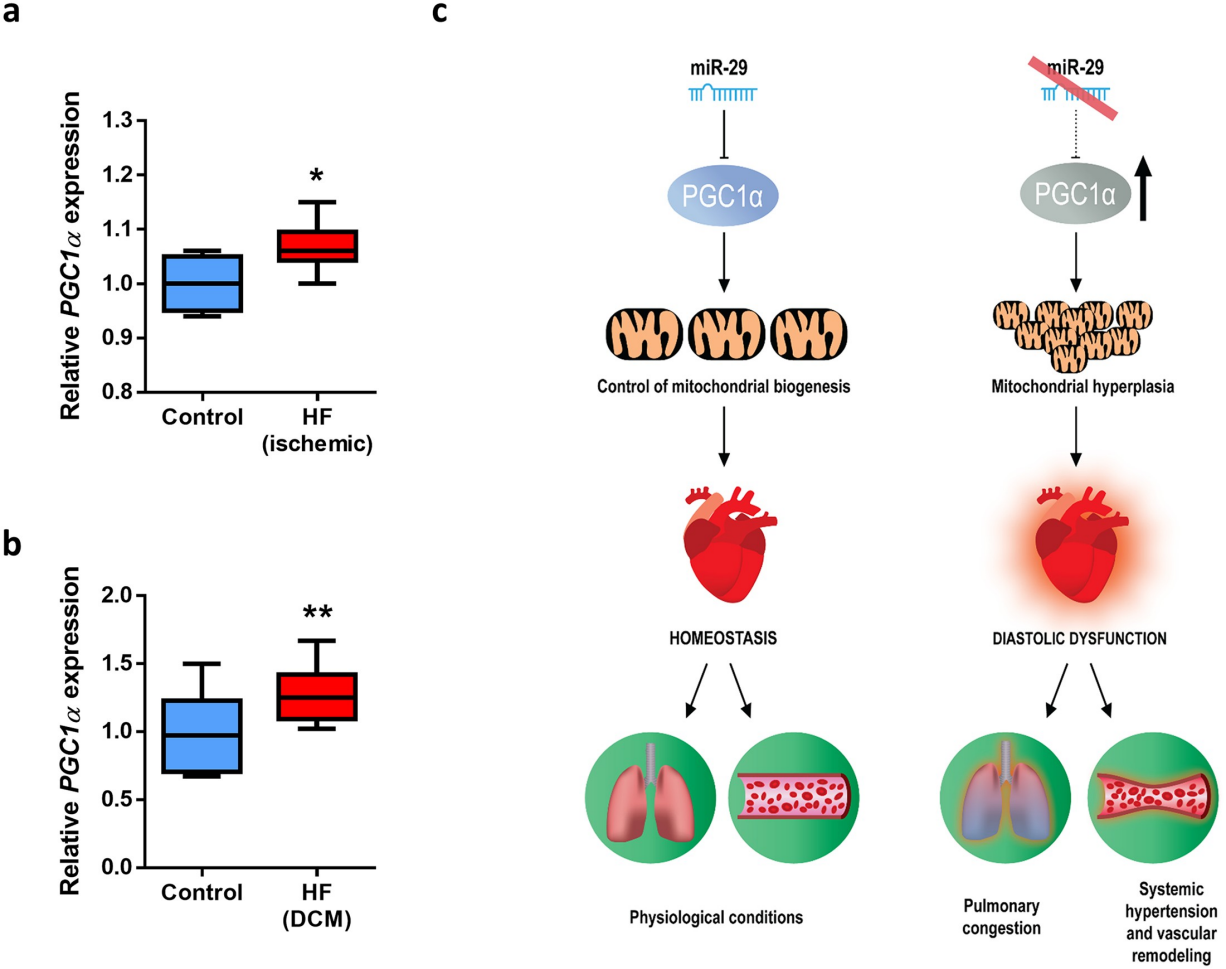
*PGC1α* is deregulated in HF patients. (A) Relative expression of *PGC1α* in cardiac biopsies from 12 patients with ischemic HF and five biopsies from the non-infarcted zone from the same individuals (GEO accession number GSE26887) [48]. (B) Relative expression of *PGC1α* in left ventricle of 16 DCM patients and 10 normal individuals (GEO accession number GSE1145). (C) Model summarizing the functional and pathological relevance of the cardiometabolic miR-29/PGC1α axis. Under physiological conditions, mature miR-29 members regulate *PGC1α* and control mitochondrial homeostasis. However, the pathologic silencing of miR-29 leads to *PGC1α* up-regulation, and the increment in the expression of this transcriptional coactivator triggers mitochondrial biogenesis and hyperplasia. Large amounts of small mitochondria in cardiomyocytes may contribute to triggering diastolic dysfunction, systemic hypertension, pulmonary congestion, and vascular remodeling, resulting in heart failure and premature death. Original raw data can be found in S1 Data file. DCM, dilated cardiomyopathy; HF, heart failure.

To extend these results to human disease, we took advantage of publicly available data of patients with cardiovascular diseases and found that in a data set derived from a study on ischemic HF patients (GEO accession number GSE26887) [48], there was evidence of a small but statistically significant increase in *PGC1α* levels in these patients (Fig 7a). Moreover, this transcriptional coactivator was also up-regulated in failing hearts from patients with diabetic dilated cardiomyopathy (DCM) (GEO accession number GSE1145) (Fig 7b). These data suggest that PGC1α expression levels may be important for cardiocirculatory homeostasis not only in mice but also in humans.

Taking these results collectively, we conclude that loss of miR-29 leads to important cardiometabolic pathological phenotypes that are mediated, at least in part, by the deregulated expression of the master metabolic regulator, PGC1α (Fig 7c).

## Discussion

We have described herein the generation and phenotypic characterization of mice deficient in the entire miR-29 family, and the functional roles of these miRNAs in cardiocirculatory and metabolic regulation. *miR-29a/b1* deficiency has been previously associated with differentiation of T helper cells and immune B cell response [49,50], neurological abnormalities [19], glucose metabolism alterations [11], liver fibrosis [14], endothelial function [51], and pulmonary hypertension [21,23]. However, our work is the first to describe a series of additional pathological abnormalities in *miR-29a/b1*^*−/−*^ mice, including urine retention, bladder dilation, ocular damage, and ataxia. More importantly, we also report the occurrence in miR-*29a/b1*^*−/−*^ mice of vascular remodeling, systemic hypertension, cardiac fibrosis, diastolic dysfunction, and pulmonary congestion, which likely contribute to triggering their premature death by HFpEF at around 7–8 months of age. Moreover, we show that these pathological alterations are ameliorated in vivo using a genetic approach based on *PGC1α* haploinsufficiency. Notably, Sassi and colleagues have recently shown that miR-29 deficiency is a cardiac-protective event in an experimental protocol of cardiac pressure overload in mice [22]. This apparent difference with our data can derive from the different experimental approaches used in both works. Sassi and colleagues used 8-week-old *miR-29a/b1*^*−/−*^ *miR-29b2/c*^*+/−*^ and *miR-29b2/c*^*−/−*^ mice and found that miR-29 deficiency protects against cardiac damage. At this young age, we were unable to identify any obvious pathological phenotype in our mice, likely because of the relatively low miR-29 expression in young mice [25]. Accordingly, and as miR-29 increases its expression with age, we hypothesize that miR-29 is required for proper cardiac function in adult mice, and its deficiency leads to cardiac alterations and premature death. We also show that *miR-29b2/c*^*−/−*^ mice exhibit minimal phenotype and functional alterations, although they also have a mild reduction in life span. Finally, we provide new evidence that double KO mice deficient in the entire miR-29 family present dramatic abnormalities, and their longevity is reduced to less than 4 weeks.

The finding of such a variety of pathological abnormalities caused by miR-29 deficiency is consistent with the pleiotropic effects of these posttranscriptional regulators, derived from their multiple and functionally diverse targets [9]. However, despite this complexity and diversity, we propose that cardiometabolic alterations, caused at least in part by *PGC1α* dysregulation, are the main life-threatening condition in *miR-29a/b1*^*−/−*^ mice. We provide evidence that the absence of miR-29 leads to elevated levels of *PGC1α*, which, owing to its functional role in mitochondrial biogenesis [52], causes a pathological accumulation of smaller organelles in mutant hearts, as assessed by both ultrastructural and molecular studies. Accordingly, transcriptome analyses revealed that important metabolic pathways (citrate cycle, oxidative phosphorylation, or glycolysis/gluconeogenesis) are deregulated in *miR-29a/b1*–null animals likely because of *PGC1α* overexpression. Mitochondria accumulation in hearts from *miR-29a/b1*^*−/−*^ mice impairs LV diastolic function, which, together with other abnormalities including cardiac fibrosis and vascular remodeling, likely leads to HFpEF and premature death. Consequently, several pathways essential for proper heart function, such as regulation of cytoskeleton, cardiac muscle contraction, gap junctions, and cAMP and calcium signaling, are also altered in mutant animals. Interestingly, these pathological phenotypes observed in *miR-29a/b1*^*−/−*^ mice are substantially improved in a *PGC1α* haploinsufficient background, indicating that the cardiovascular abnormalities in *miR-29a/b1*^*−/−*^ mice are mainly driven by this transcriptional coactivator.

Consistent with our findings, mitochondrial fragmentation triggers HF in mice [33], which further supports that *PGC1α* and mitochondrial biogenesis could be important for the pathogenesis of this disease. In fact, mice overexpressing *PGC1α* specifically in the heart show a dramatic increase in the number of cardiac mitochondria and profound alterations in mitochondrial ultrastructure, which result in the development of a marked cardiomyopathy [45,46]. Additionally, the size and the morphology of mitochondria, which are regulated by fusion/fission events, are also instrumental to their correct function [53]. Notably, our results show that mitochondria of *miR-29a/b1*–deficient mice are smaller compared with those of wild-type animals. Accordingly, we propose that the altered structure of mitochondria of *miR-29a/b1*–mutant mice also contributes to impairing cardiac function, although further long-term studies involving conditional miR-29–mutant mice will be helpful to clarify the in vivo roles of miR-29 in HF and the relative relevance of different cell types to the pathogenesis of this disease. We also assume that besides the cardiometabolic alterations reported herein for miR-29–deficient mice, these animals may have additional metabolic abnormalities, which could result from the dysregulation of miR-29–modulated molecular pathways outside the cardiovascular system. Thus, and in agreement with previous studies [11], we have found that *miR-29a/b1*^*−/−*^ mice exhibit hyperglycemia and reduced insulin concentration, two common features of diabetic patients.

Finally, and in relation to the putative impact of these studies on human disease, a recent study has revealed that miR-29b is reduced in end-stage HF patients [54]. Moreover, our findings that mice deficient in *miR-29a/b1* develop cardiovascular abnormalities and that both mutant mice and HF patients exhibit *PGC1α* overexpression suggest that miRNA replacement therapies could be beneficial for these patients, as proposed for other pathologies associated with miRNA deregulation [15,55,56].

In summary, the generation of *miR-29*–deficient mice has allowed us to identify the in vivo relevance of this family of miRNAs in cardiometabolic regulation. miR-29 is essential to maintain cardiovascular function through the transcriptional coactivator PGC1α, which in turn controls mitochondrial biogenesis and homeostasis. The absence of miR-29 expression triggers cardiovascular fibrosis, systemic hypertension, diastolic dysfunction, and HF that can be extensively rescued by genetic approaches involving heterozygous loss of *PGC1α*, which is thus validated as an in vivo functional target of miR-29. Overall, this work provides novel mechanistic insights into the relationship of miRNAs and cardiometabolic diseases, opening the possibility of clinical approaches to these pathologies based on targeting the newly identified regulatory circuitry involving miR-29 and PGC1α.

## Materials and methods

### Ethics statement

All animal procedures were approved and performed in accordance with the guidelines of the Committee for Animal Experimentation at the Universidad de Oviedo. For tissue collection, mice were given a lethal dose of ketamine/xylazine intraperitoneally.

### Mouse models

For the generation of miR-29 KO mice, we obtained two ES cell lines with targeted deletions in the *miR-29a/b1* and *miR-29b2/c* clusters from Dr. Haydn M. Prosser [57]. Mutant ES cells were screened by Southern blot and used to generate chimeric mice that were then bred with C57BL/6 animals to generate heterozygous mice. After intercrossing heterozygous mice from each of the strains, we generated *miR-29a/b1*^*−/−*^ and *miR-29b2/c*^*−/−*^ animals at the expected Mendelian ratio. We used genomic DNA from tail biopsies, extracted by alkaline lysis, for PCR genotyping using Platinum Taq DNA polymerase (Invitrogen, Waltham, MA) under the following conditions: denaturation at 95 °C for 15 seconds, annealing at 61 °C for 15 seconds, and extension at 72 °C for 35 seconds; 35 cycles for both clusters. *PGC1α*-deficient mice were provided by Dr. Bruce M. Spiegelman [43]. Genotyping conditions were as follows: denaturation at 95 °C for 15 seconds, annealing at 55 °C for 30 seconds, and extension at 72 °C for 30 seconds; 35 cycles. *Eln*-null mice were provided by Dr. Dean Li [58]. Genotyping conditions were as follows: denaturation at 95 °C for 30 seconds, annealing at 62 °C for 20 seconds, and extension at 72 °C for 45 seconds, during 10 cycles; then, denaturation at 95 °C for 30 seconds, annealing at 57 °C for 20 seconds, and extension at 72 °C for 45 seconds, during 25 cycles. All primers used for genotyping are listed in S2 Table.

### Animal studies

Mice were weighted once a week for longevity experiments. The angiotensin II–induced cardiac fibrosis protocol was performed as described [59]. Angiotensin II (3 μg × Kg^−1^ × min^−1^) (Sigma, St. Louis, MO, ref. A9525) was administered subcutaneously via Alzet (Cupertino, CA) osmotic minipumps (Model 1007D) for 6 days. For adipose tissue evaluation, mice were killed and adipose depots were harvested and weighted.

### Histological analysis

Tissues were fixed in 4% paraformaldehyde in phosphate-buffered saline (PBS) and stored in 50% ethanol. Fixed tissues were embedded in paraffin by standard procedures. Blocks were sectioned (5 μm) and hematoxylin–eosin (HE), orcein for elastic fibers, Masson’s and Gomori’s Trichrome, or Verhoeff–Van Gieson elastic staining were performed. All tissues were examined by a pathologist (Dr. M.T. Fernández-García) in a blinded fashion. For collagen quantification, we used a FIJI plugging provided by A. M. Nistal (Servicios Científico-Técnicos, Universidad de Oviedo) [60]. For adipose tissue evaluation, portions of gonadal fat pads were processed as described above. The number of adipocytes and their mean diameter were determined in four tissue sections per mouse by computer-assisted image analysis. For adipocyte size, 100 cells per section were measured. For the number of adipocytes per area, we counted the cells included in a counting frame of 272,613.37 μm^2^. For angiotensin II–induced cardiac fibrosis protocol, after 28 days of angiotensin II infusions, hearts were harvested, fixed in paraformaldehyde 4% in PBS, embedded in paraffin, sectioned every 200 μm, and stained with Gomori’s Trichrome. Fibrotic lesions were classified in three categories: Small (≤200 μm), medium (>200 μm and ≤400 μm), and large (>400 μm), depending on the number of correlative heart sections where lesions appeared. Fibrotic extension was graded from 0 to 4 according to a clinical score. Grade 0: no fibrotic areas; grade 1: less than 25% of myocardium affected; grade 2: from 26% to 50% of myocardium affected; grade 3: from 51% to 75% of myocardium affected; and grade 4: more than 76% of myocardium affected. Pulmonary congestion was graded 0, 0.5, 1, 2, or 3 for no, very mild, mild, moderate, or severe injury, respectively, according to previously published pathological criteria [61,62].

### Immunohistochemistry

Immunohistochemistry was performed on paraffin 5-μm-thick sections. Antigen retrieval was carried out by heating 20 minutes at 95 °C using EnVision LEX Target Retrieval Solution, Low pH (Dako, Santa Clara, CA). Endogen peroxidase activity was quenched by incubation in Peroxidase-Blocking Solution (Dako, Santa Clara, CA). Each incubation step was carried out at room temperature and followed by three sequential washes in TBS-T. To block nonspecific binding, tissues were incubated for 60 minutes in Protein-Block Serum-Free (Dako, Santa Clara, CA). Then, samples were incubated at room temperature 5 minutes with anti–von Willebrand factor (VWF, Dako, Santa Clara, CA, ref. GA527, prediluted) or 10 minutes with anti–α-smooth muscle actin (SMA, Sigma, St. Louis, MO, ref. A5228, 1:100), followed by incubations with Liquid DAB (Dako, Santa Clara, CA) for 10 minutes. Control immunohistochemical staining was performed following the same procedure but omitting the primary antibody. Procedures were performed in Autostainer Plus for IHC (Dako, Santa Clara, CA) and PT Link (Dako, Santa Clara, CA). Finally, slides were counterstained, dehydrated, and mounted.

### Blood, serum, and plasma parameters

Animals fed a regular diet were fasted overnight and used for measurements of different blood and plasma parameters. Blood glucose was measured with an Accu-Chek glucometer (Roche, Basel, Switzerland) using blood from the tail vein. Blood was extracted from submandibular sinus after anesthetizing mice with isoflurane. Serum was obtained by centrifugation at 3,000*g* and 4 °C, after keeping the blood 30 minutes at room temperature. The supernatant was collected and stored at −80 °C until analysis. For insulin measurement, we used Millipore (Burlington, MA) ELISA kit (EZRMI-13K) according to the manufacturer’s instructions. Creatinine quantitation was performed with a Flex reagent cartridge and using the Dimension RxL clinical chemistry system (Siemens Healthcare, Erlangen, Germany). For double KO mice, blood glucose measurements were performed using blood from nonfasted animals, given the impossibility of fasting infant mice.

### Cardiovascular studies

Cardiac functions and left ventricle dimensions were analyzed by transthoracic echocardiography using a Vevo 2100 system and a 30-MHz probe (Visualsonics, Toronto, Canada). Measurements were taken in a blinded fashion, with mice placed on a heating pad under light anesthesia (1.5%–2% isoflurane in oxygen, provided through a facial mask). Measurements were repeated between 2 and 5 times per animal, and the average was used for downstream analysis. Blood pressure was measured with a noninvasive automated tail-cuff device (Visitech System BP2000, Sunderland, UK) in conscious mice. Measurements were taken on a daily basis for 2 weeks at the same time in the morning. To improve accuracy, the first 10 of 20 measurements were discarded. The interquartile range (IQR) was calculated (the difference between quartiles 3 and 1) and data points 1.5 × IQR below the first quartile (Q1) or 1.5 × IQR above the third quartile (Q3) were discarded as outliers. Values over 200 mm Hg in pressure measurements were considered artifacts and therefore excluded. Daily median values of the remaining measurements for individual mice were used for analysis. For lung weight/body weight ratio, mice were weighed and killed. Subsequently, left lungs were perfused with saline solution, harvested, and weighed.

### Real-time quantitative PCR

cDNA was synthesized from 1 μg of Trizol-extracted (Life Technologies, Carlsbad, CA) total RNA using QuantiTect Reverse Transcription Kit (Qiagen, Hilden, Germany) following the manufacturer’s instructions. Quantitative cDNA amplification was carried out in triplicate for each sample in a final reaction volume of 10 μL, using 4.5 μL of cDNA (1/10 diluted), 5 μL Power SYBR Green Master Mix (Applied Biosystems, Foster City, CA), and 0.5 μL of 10-μM specific oligonucleotides for the gene of interest (Sigma, St. Louis, MO), in an ABI Prism 7300 sequence detector system with standard settings. Relative expression was calculated as RQ = 2^−ΔΔCt^. The oligonucleotides are indicated in S2 Table. As an internal control, gene expression was normalized to the mouse *Actb*, *Gapdh*, or 18S genes. miR-29 detection was performed using Taqman miRNA expression assays (Applied Biosystems, Foster City, CA) for the double KO mice experiments or Taqman Advance miRNA expression assays (Applied Biosystems, Foster City, CA) for the single KO mice experiments. As an internal control, miR-29 expression was normalized against snoRNA202 (Taqman miRNA Assays) or 18S (Taqman Advance miRNA assays). All protocols were carried out according to the manufacturer’s instructions.

### Western blotting

Protein lysates were prepared in RIPA buffer and equal amounts of total proteins were loaded onto SDS-polyacrylamide gels. After electrophoresis, gels were electrotransferred onto nitrocellulose membranes and incubated overnight with the different primary antibodies used. Finally, blots were incubated for 1 hour, with corresponding secondary antibodies conjugated with horseradish peroxidase (HRP) to develop immunoreactive bands with Immobilon Western Chemiluminescent HRP substrate (Merck, Darmstadt, Germany) in a LAS-3000 Imaging system (Fujifilm, Tokyo, Japan). The primary antibodies used in this study were anti-PGC1α 1:500 (Santa Cruz, Dallas, TX, ref. H-300), 1:1,000 anti-PPARγ (Cell Signaling, Danvers, MA, clone 81B8), 1:5,000 anti-Gapdh-peroxidase (Sigma, St. Louis, MO, ref. G9295), and anti-α-tubulin 1:10,000 (Sigma, St. Louis, MO, clone B-5-1-2). Densitometry was performed using Multi Gauge software (Fujifilm, Tokyo, Japan).

### TEM

Hearts were harvested from mice and immediately fixed in 3% glutaraldehyde in 0.1 M sodium cacodylate (pH 7.2) overnight. Then, the samples were transferred to a storage solution with 1.5% glutaraldehyde in 0.1 M sodium cacodylate (pH 7.2) until further use. The pieces were processed for Durcapan ACM (Fluka BioChemika, Munich, Germany) resin embedding as follows: previously fixed samples were immersed in 5% sucrose in sodium cacodylate buffer (0.1 M; pH 7.4) for 3×10 minutes, postfixed with 1% osmium tetroxide in sodium cacodylate buffer for 3 hours in darkness, and washed in sodium cacodylate buffer for 3×10 minutes. Then, samples were dehydrated with increasing acetone concentrations: 30% for 10 minutes, 60% for 10 minutes, 90% for 10 minutes, and 100% for 3×10 minutes. The dehydrated pieces were then immersed in mixtures of anhydrous acetone with increasing resin concentrations (1:1, 1:2) for 30 minutes, and then in pure resin (at 37 °C for 12 hours following 60 °C for 24 hours). Sections were obtained with a Reichert Jung Ultracut E ultramicrotome. Semi-thin sections (1 μm) were stained with toluidine blue and examined with a light microscope. Then, ultrathin sections (80 nm) were obtained from selected areas of the semi-thin sections, stained with uranyl acetate (2 minutes), washed with distilled water and stained with lead citrate (2 minutes), and finally washed quickly with distilled water. Finally, sections were examined and photographed with a JEOL JEM-1011 HR (Universidad de Valladolid) electron microscope and JEOL JEM-1011 (Servicios Científico-Técnicos, Universidad de Oviedo). Six fields per sample were analyzed. ImageJ software [63] was used for the quantitative analysis of the mitochondrial number and area.

### Mitochondrial DNA copy number quantification

We quantified mtDNA by real-time qPCR using an ABI PRISM 7300 Sequence Detector System (Applied Biosystems, Foster City, CA) and SYBR PCR Master Mix (Applied Biosystems, Foster City, CA). A total of 40 ng of total DNA was used as a template to amplify with specific oligonucleotides for the mitochondrial 16s gene (mt-16s). We calculated the mtDNA copy number per cell using *Ucp2* as a reference from the nuclear genome. Oligonucleotide sequences can be found in S2 Table.

### Computed tomography scan

Computed tomography (CT) scans were performed on unconscious mice using Argus PET/CT (Sedecal, Servicios Científico-Técnicos, Universidad de Oviedo, Spain). For image analysis we used Amide software.

### miR-29 and PGC1a expression in mouse tissues

miRNA and mRNA expression data from mouse postnatal day 0 tissues were retrieved from the Encyclopedia of DNA elements (ENCODE) [64]. For miRNA analysis, read alignments in BAM format were downloaded from ENCODE, and the read counts mapping to the mature miRNAs were calculated using HTSeq python package [65] with options “*-m intersection-strict—nonunique all -a 0*” (to account for multiple-copy miRNAs like miR-29b) and the mouse miRBase miRNA annotation release 22 [66]. All further analysis was done using R-language [67]. For plotting, miRNA read counts were normalized to counts per million (CPM) reads using the total number of reads mapping to miRNAs. For gene expression analysis, gene quantifications in tabular format were downloaded from ENCODE, and TPM (transcripts per million) reads values were used directly for plotting using R-language. Metadata and accession numbers of the data used in this analysis can be found in S3 Table.

### Transcriptome studies

Trizol-extracted total RNA from heart biopsies of three wild-type, *miR-29a/b1*^*−/−*^, and *miR-29b2/c*^*−/−*^ mice was paired-end sequenced by BGI (Shenzhen, China) using strand-specific polyA-selected library preparation in a BGI-500 sequencer. Data are available at the European Nucleotide Archive (ENA) with accession number ERP110123. Clean (quality-filtered, adaptor-trimmed) raw reads provided by BGI were further processed using Salmon software [68] to calculate transcript abundances. Differential expression analysis was performed using R-package DESeq2 [69], with gene-aggregated counts calculated by Salmon. For miR-29 target analysis, conserved miRNA predictions from mouse TargetScan [70] were crossed with the differentially expressed genes identified by DESeq2 using R-language. A comprehensive list of differentially regulated genes in either *miR-29a/b1*^*−/−*^ or *miR-29c/b2*^*−/−*^ mice compared with wild type can be found in S1 Table. Pathway analysis was done using the R-package pathfindR [71] and the KEGG database [72]. Heat map plots were generated using R-language employing DESeq2 log-transformed counts gene-wise normalized (z-score). Venn diagrams were generated with R-package nVennR [73] using differentially expressed genes defined by DESeq2.

### Statistical analysis

Unless otherwise specified, bar plots represent the mean and the standard error of the mean (SEM). Differences between mean values were analyzed using the two-tailed Student *t* test (continuous variables) or Mann–Whitney test (discrete variables) for comparisons between two groups and ANOVA (continuous variables) or Kruskal–Wallis (discrete variables) with post hoc testing (uncorrected Fisher’s LSD or Dunn’s tests, respectively), except in cases that indicate the use of a different statistical test. Student *t* test was Welch-corrected for variables with different variances. *p* < 0.05 was considered significant, and statistically significant differences are shown with asterisks (*p < 0.05, **p < 0.01, and ***p < 0.001). All comparisons between wild-type and mutant mice were performed in animals of similar age. Experimental conditions were blinded and randomized, and no statistical method was used to predetermine sample size. Differences between groups were assayed using Microsoft Excel, SPSS, and GraphPad Prism. For blood pressure measurements, custom R scripts were used for statistical analysis. Each parameter was transformed by natural logarithms to normalize data distributions, and differences between genotypes were assessed by a linear regression model taking into account the technical replicates of the experiments, as well as the age and sex of the mice. Human *PCG1α* expression data were extracted from previously reported comparative data sets of transcriptomic analyses in cardiac tissue (GEO accession numbers GSE26887 and GSE1145) [48].

## Supporting information

S1 Fig Generation of miR-29–deficient mice. (A) Schematic representation of wild-type and puroΔtk alleles, and the genotyping strategy. The replacement of each miR-29 cluster was performed following a homologous recombination strategy using the puroΔtk vector. (B) The generation of puroΔtk allele in both miR-29 clusters was confirmed by Southern blot of genomic DNA from heterozygous ES cells. Original raw data can be found in S1 Data file. ES, embryonic stem.
(TIF)

S2 Fig Metabolic alterations in miR-29–deficient mice. (A) Gonadal white adipose tissue (WAT) and interscapular brown adipose tissue (BAT) fat mass represented as a percentage of total body weight of wild-type (*n* = 3), *miR-29a/b1*^*−/−*^ (*n* = 3), and *miR-29b2/c*^*−/−*^ (*n* = 2) mice. (B) Representative picture of gonadal fat depots in wild-type, *miR-29a/b1*^*−/−*^, and *miR-29b2/c*^*−/−*^ mice. (C) HE sections of gonadal WAT of wild-type, *miR-29a/b1*^*−/−*^, and *miR-29b2/c*^*−/−*^ mice (original magnification: ×20, scale bar: 20 μm). (D) Mean adipocyte number per counting area in gonadal WAT of wild-type (*n* = 3), *miR-29a/b1*^*−/−*^ (*n* = 3), and *miR-29b2/c*^*−/−*^ (*n* = 2) mice. (E) Mean adipocyte size in gonadal WAT of wild-type (*n* = 3), *miR-29a/b1*^*−/−*^ (*n* = 3), and *miR-29b2/c*^*−/−*^ (*n* = 2) mice. Original raw data can be found in S1 Data file. BAT, brown adipose tissue; HE, hematoxylin–eosin; WAT, white adipose tissue.
(TIF)

S3 Fig Body weight curve of *miR-29b2/c*^*−/−*^ mice. Body weight curves of wild-type (*n* = 8) and *miR-29b2/c*^*−/−*^ (*n* = 14) male mice (*p* < 0.05 at 41 and 42 weeks, two-tailed multiple Student *t* test, Bonferroni-corrected). Original raw data can be found in S1 Data file.
(TIF)

S4 Fig miR-29 expression patterns in mice. (A) miR-29 expression patterns from mouse postnatal day 0 tissues (ENCODE; average of duplicates if applicable). The dotted line indicates the mean expression value across all tissues. (B) Linear regression analysis between the expression of miR-29 family members from mouse postnatal day 0 tissues. Original raw data can be found in S1 Data file. CPM, counts per million; ENCODE, Encyclopedia of DNA Elements.
(TIF)

S5 Fig miR-29 relative expression in *miR-29a/b1*^*−/−*^, *miR-29b2/c*^*−/−*^, and double KO mice. (A) Relative expression of miR-29 family members in heart, liver, and kidney samples from wild-type (*n* = 6), *miR-29a/b1*^*−/−*^ (*n* = 3), and *miR-29b2/c*^*−/−*^ (*n* = 3) mice. (B) Relative expression of miR-29 family members in heart, lung, and kidney samples from wild-type (*n* = 3) and double KO (*n* = 3) mice. Original raw data can be found in S1 Data file. KO, knock-out.
(TIF)

S6 Fig Insulin measurement in miR-29–deficient mice. Levels of insulin measured by ELISA in serum from wild-type (*n* = 10), *miR-29a/b1*^*−/−*^ (*n* = 8), and *miR-29b2/c*^*−/−*^ (*n* = 8) mice. Original raw data can be found in S1 Data file.
(TIF)

S7 Fig Metabolic profiling in mice deficient in miR-29. (A) Expression of key metabolic genes measured by RT-qPCR in livers from wild-type (*n* = 5), *miR-29a/b1*^*−/−*^ (*n* = 5), and *miR-29b2/c*^*−/−*^ (*n* = 4) mice. (B) Expression of key metabolic genes analyzed by RT-qPCR in livers from wild-type (*n* = 5) and double KO (*n* = 6) mice. Original raw data can be found in S1 Data file. RT-qPCR, quantitative reverse transcription PCR.
(TIF)

S8 Fig Two-dimensional echocardiography of *miR-29a/b1*^*−/−*^ mice. Quantification of structural parameters: (A) the left ventricular posterior wall (LVPW), (B) interventricular septum (IVS) thickness, and (C) left ventricular internal diameter (LVID), corrected by tibia length of wild-type (*n* = 10) and *miR-29a/b1*^*−/−*^ (*n* = 9) mice. Quantification of functional parameters: (D) ejection fraction in wild-type (*n* = 10) and *miR-29a/b1*^*−/−*^ (*n* = 8) mice. Original raw data can be found in S1 Data file. IVS, interventricular septum; LVID, left ventricular internal diameter; LVPW, left ventricular posterior wall.
(TIF)

S9 Fig Heart metabolic switch from fatty acids to glucose in *miR29a/b1*^*−/−*^ mice. Expression of key metabolic genes measured by RT-qPCR in wild-type (*n* = 5) and *miR-29a/b1*^*−/−*^ (*n* = 12) mice. Original raw data can be found in S1 Data file. RT-qPCR, quantitative reverse transcription PCR.
(TIF)

S10 Fig Lack of renal failure in *miR-29a/b1*^*−/−*^ mice. (A) Serum creatinine levels in wild-type (*n* = 3) and *miR-29a/b1*^*−/−*^ (*n* = 3) mice. (B) HE sections of wild-type and *miR-29a/b1*^*−/−*^ mice (original magnification: ×20, scale bar: 100 μm). Original raw data can be found in S1 Data file. HE, hematoxylin–eosin.
(TIF)

S11 Fig Transcriptome analysis in miR-29 mutant mice. (A) Venn diagrams of differentially expressed genes (adjusted *p*-value < 0.01 and absolute log2 fold change > 0.8) in *miR-29a/b1*^*−/−*^ and *miR-29b2/c*^*−/−*^ mice compared with wild-type. (B) Venn diagram of differentially expressed miR-29–predicted target genes (adjusted *p*-value < 0.01 and absolute log2 fold change > 0.8) in *miR-29a/b1*^*−/−*^ and *miR-29b2/c*^*−/−*^ mice compared with wild-type. (C) Heat map plot of z-score normalized TPM of miR-29 predicted target genes significantly up-regulated in either in *miR-29a/b1*^*−/−*^ and *miR-29b2/c*^*−/−*^ mice compared with wild type. Original raw data can be found in S1 Data file. TPM, transcripts per million.
(TIF)

S12 Fig *Eln* expression is up-regulated in *miR-29a/b1*^*−/−*^ mice, but its haplodeficiency does not improve the life span of mutant animals. (A) *Eln* expression in hearts from wild-type (*n* = 4) and *miR-29a/b1*^*−/−*^ (*n* = 4) mice. (B) Kaplan–Meier survival plot of *miR-29a/b1*^*−/−*^ *Eln*^*+/+*^ (*n* = 10) and *miR-29a/b1*^*−/−*^ *Eln*^*+/−*^ (*n* = 21) mice. Original raw data can be found in S1 Data file. *Eln*, elastin.
(TIF)

S13 Fig miR-29 transcriptionally regulates *PGC1α* expression. Sequence and putative miR-29–binding sites of *PGC1α*. All family members regulate *PGC1α* expression. Original raw data can be found in S1 Data file.
(TIF)

S14 Fig *PGC1α* expression in miR-29–deficient mice. RT-PCRs of *PGC1α* in (A) liver and kidney tissues from wild-type (*n* = 4) and *miR-29a/b1*^*−/−*^ (*n* = 4) mice and (B) hearts from wild-type (*n* = 3) and *miR-29c/b2*^*−/−*^ (*n* = 3) mice. Original raw data can be found in S1 Data file. RT-PCR, quantitative reverse transcription PCR.
(TIF)

S15 Fig *PGC1α* expression in ENCODE data. (A) *PGC1α* expression levels represented as transcripts per million (TPM) in different tissues from postnatal day 0 mice (average and standard error of the mean). (B) Pearson correlation between *PGC1a* and total miR-29 expression levels (sum of miR-29a, -b, and -c). Original raw data can be found in S1 Data file. CPM, counts per million; TPM, transcripts per million.
(TIF)

S16 Fig Protein levels of *Pparγ* in *miR-29a/b1*^*−/−*^ mice. Western blot analysis using antibodies against *Pparγ* in protein extracts of hearts from wild-type (*n* = 3), *miR-29a/b1*^*−/−*^ *PGC1α*^+/+^ (*n* = 3), and *miR-29a/b1*^*−/−*^ *PGC1α*^*+/−*^ (*n* = 3). *Gapdh* detection in the same blot was used as loading control. Original raw data can be found in S1 Data file.
(TIF)

S17 Fig Pathway analysis of gene expression changes in miR-29 mutant mice. (A) “Bubble plot” showing pathways significantly enriched (adjusted enrichment *p*-value < 0.01; red color scale) in differentially expressed genes (DEG; size of the points) in *miR-29a/b1*^*−/−*^ mice compared with wild-type mice. (B) Heat map plots of relevant pathways showing the z-score transformed TPM in hearts from wild-type, *miR-29a/b1*^*−/−*^, and *miR-29c/b2*^*−/−*^ mice. Original raw data can be found in S1 Data file. DEG, differentially expressed gene; TPM, transcripts per million.
(PDF)

S18 Fig Mitochondrial alterations in *miR-29a/b1*^*−/−*^ mice. Quantification of mean mitochondrial size in wild-type (six photographs from two different mice), *miR-29a/b1*^*−/−*^ *PGC1α*^*+/+*^ (22 photographs from four different mice), and *miR-29a/b1*^*−/−*^ *PGC1α*^*+/−*^ (22 photographs from three different mice) animals. Original raw data can be found in S1 Data file.
(TIF)

S19 Fig Rescue of cardiac function in *miR-29a/b1*^*−/−*^ mice after *PGC1α* heterozygosis. (A) Mean blood pressure values from wild-type (*n* = 10), *miR-29a/b1*^*−/−*^ *PGC1α*^*+/+*^ (*n* = 8), and *miR-29a/b1*^*−/−*^ *PGC1α*^*+/−*^ (*n* = 6) mice. (B) Quantification of DT of early filling fraction in wild-type (*n* = 10), *miR-29a/b1*^*−/−*^ *PGC1α*^+/+^ (*n* = 9), and *miR-29a/b1*^*−/−*^ *PGC1α*^*+/−*^ (*n* = 6) mice. (C) Quantification of IVRT in wild-type (*n* = 10), *miR-29a/b1*^*−/−*^ *PGC1α*^*+/+*^ (*n* = 9), and *miR-29a/b1*^*−/−*^ *PGC1α*^*+/−*^ (*n* = 6) mice. Original raw data can be found in S1 Data file. DT, deceleration time; IVRT, isovolumetric relaxation time.
(TIF)

S1 Table Differentially expressed genes in transcriptome analysis. Full list of differentially expressed genes (adjusted *p*-value < 0.05) in *miR-29a/b1*^*−/−*^ or *miR-29c/b2*^*−/−*^ compared with wild-type mice, indicating the number of conserved miR-29 binding sites, if applicable.
(XLSX)

S2 Table List of oligonucleotides used in this work. (XLSX)

S3 Table Accession numbers and metadata of ENCODE samples used in this work. (XLSX)

S1 Video Ataxia in *miR-29a/b1*^*−/−*^ mice. Video showing the movement of WT (left) and *miR-29a/b1*^*−/−*^ mice (right). WT, wild-type.
(MP4)

S1 Data Excel file containing the underlying numeric data used in this work. (XLSX)

## Abbreviations

AMPK: AMP-activated protein kinase
cAMP: cyclic AMP
CPM: counts per million
CT: computed tomography
DCM: dilated cardiomyopathy
DT: deceleration time
E/A: early and late diastolic filling velocities ratio
Eln: elastin
ENCODE: Encyclopedia of DNA Elements
ES: embryonic stem
HE: hematoxylin–eosin
HF: heart failure
HFpEF: HF with preserved ejection fraction
IVRT: isovolumetric relaxation time
KEGG: Kyoto Encyclopedia of Genes and Genomes
KO: knock-out
LV: left ventricular
miRNA: microRNA
mtDNA: mitochondrial DNA
ncRNA: noncoding RNA
NS: nonsignficant
RT-qPCR: quantiative reverse transcription PCR
SMA: α-smooth muscle actin
TEM: transmission electron microscopy
TPM: transcripts per million
VWF: von Willebrand factor
