# Supplementary figures and images for: The microRNA-29/PGC1α regulatory axis is critical for metabolic control of cardiac function
Source: PLoS Biol. 2018 Oct 22;16(10):e2006247. doi: 10.1371/journal.pbio.2006247 (PMC6211751; doi:10.1371/journal.pbio.2006247)

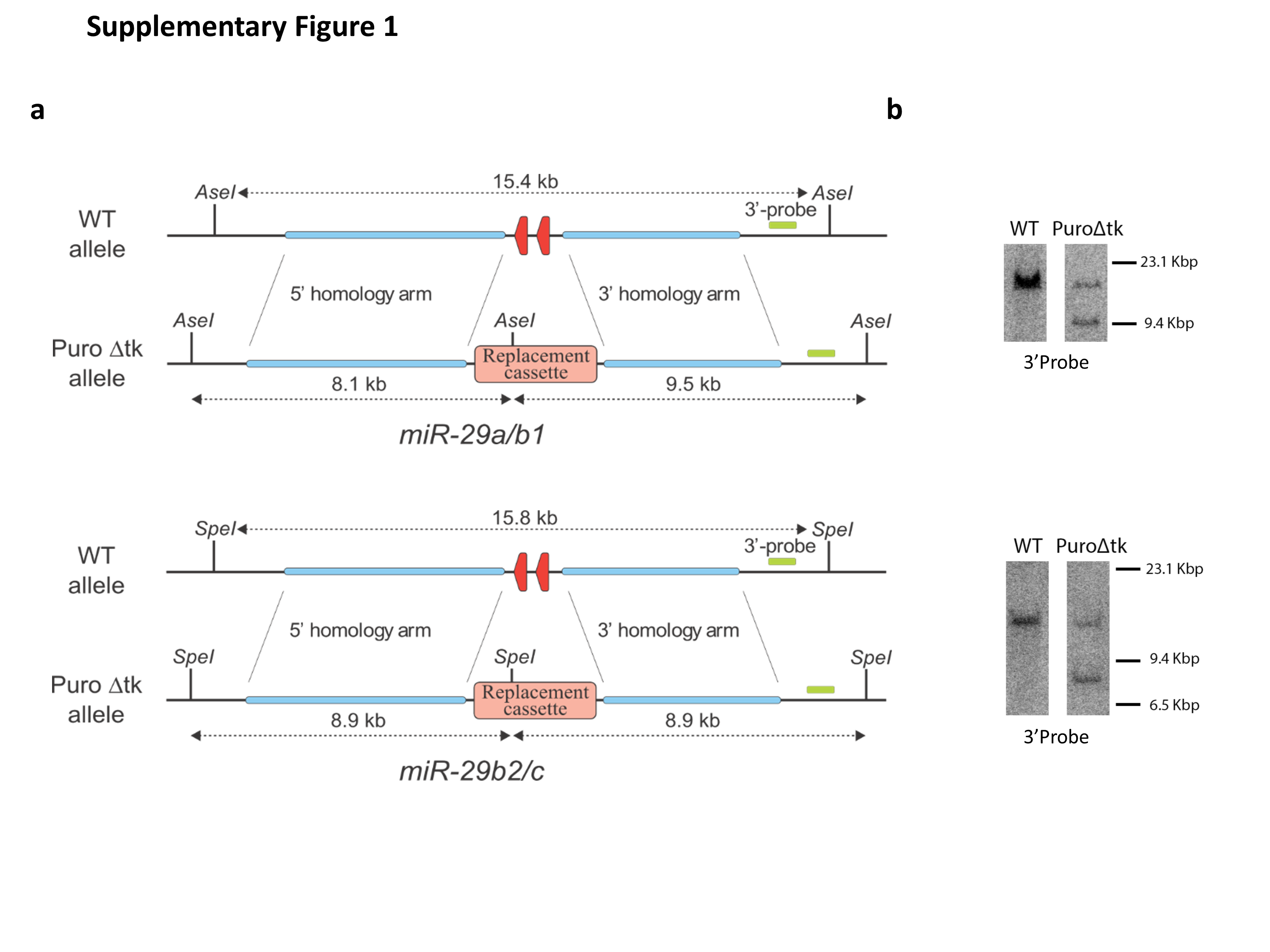

Supplement: S1 Fig — (A) Schematic representation of wild-type and puroΔtk alleles, and the genotyping strategy. The replacement of each miR-29 cluster was performed following a homologous recombination strategy using the puroΔtk vector. (B) The generation of puroΔtk allele in both miR-29 clusters was confirmed by Southern blot of genomic DNA from heterozygous ES cells. Original raw data can be found in S1 Data file. ES, embryonic stem. (TIF) [file pbio.2006247.s001.TIF]

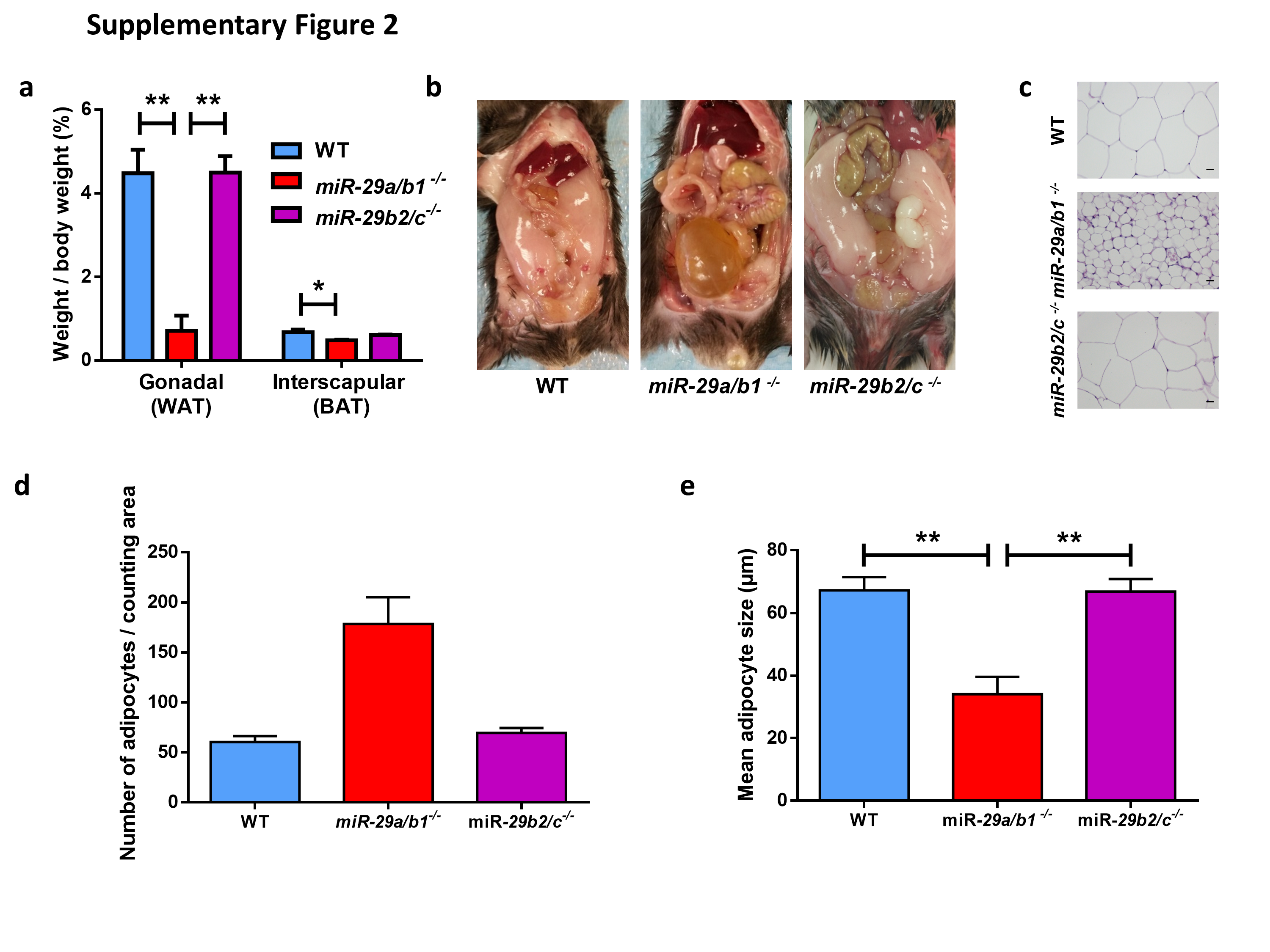

Supplement: S2 Fig — (A) Gonadal white adipose tissue (WAT) and interscapular brown adipose tissue (BAT) fat mass represented as a percentage of total body weight of wild-type (n = 3), miR-29a/b1−/− (n = 3), and miR-29b2/c−/− (n = 2) mice. (B) Representative picture of gonadal fat depots in wild-type, miR-29a/b1−/−, and miR-29b2/c−/− mice. (C) HE sections of gonadal WAT of wild-type, miR-29a/b1−/−, and miR-29b2/c−/− mice (original magnification: ×20, scale bar: 20 μm). (D) Mean adipocyte number per counting area in gonadal WAT of wild-type (n = 3), miR-29a/b1−/− (n = 3), and miR-29b2/c−/− (n = 2) mice. (E) Mean adipocyte size in gonadal WAT of wild-type (n = 3), miR-29a/b1−/− (n = 3), and miR-29b2/c−/− (n = 2) mice. Original raw data can be found in S1 Data file. BAT, brown adipose tissue; HE, hematoxylin–eosin; WAT, white adipose tissue. (TIF) [file pbio.2006247.s002.TIF]

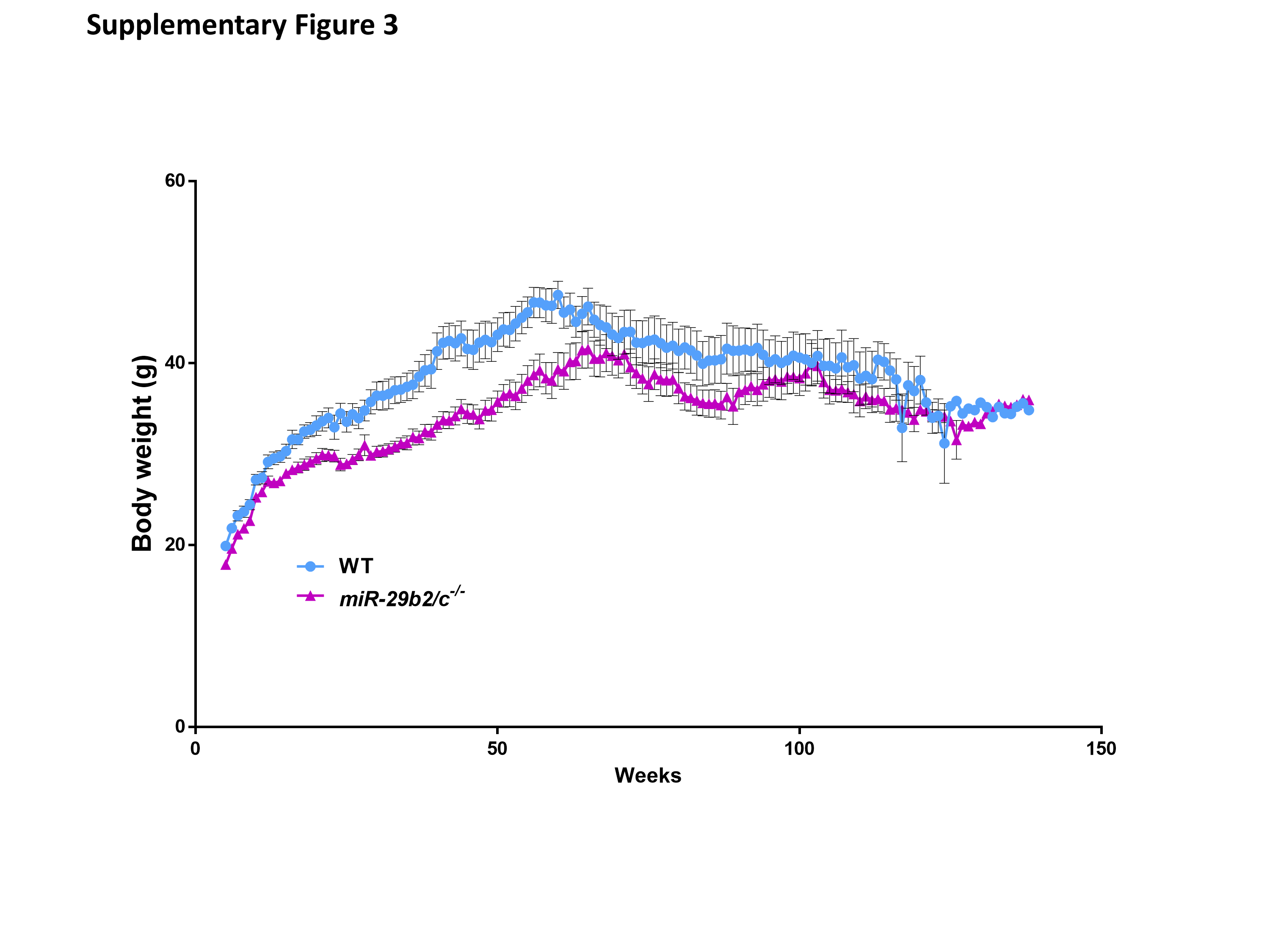

Supplement: S3 Fig — Body weight curves of wild-type (n = 8) and miR-29b2/c−/− (n = 14) male mice (p < 0.05 at 41 and 42 weeks, two-tailed multiple Student t test, Bonferroni-corrected). Original raw data can be found in S1 Data file. (TIF) [file pbio.2006247.s003.TIF]

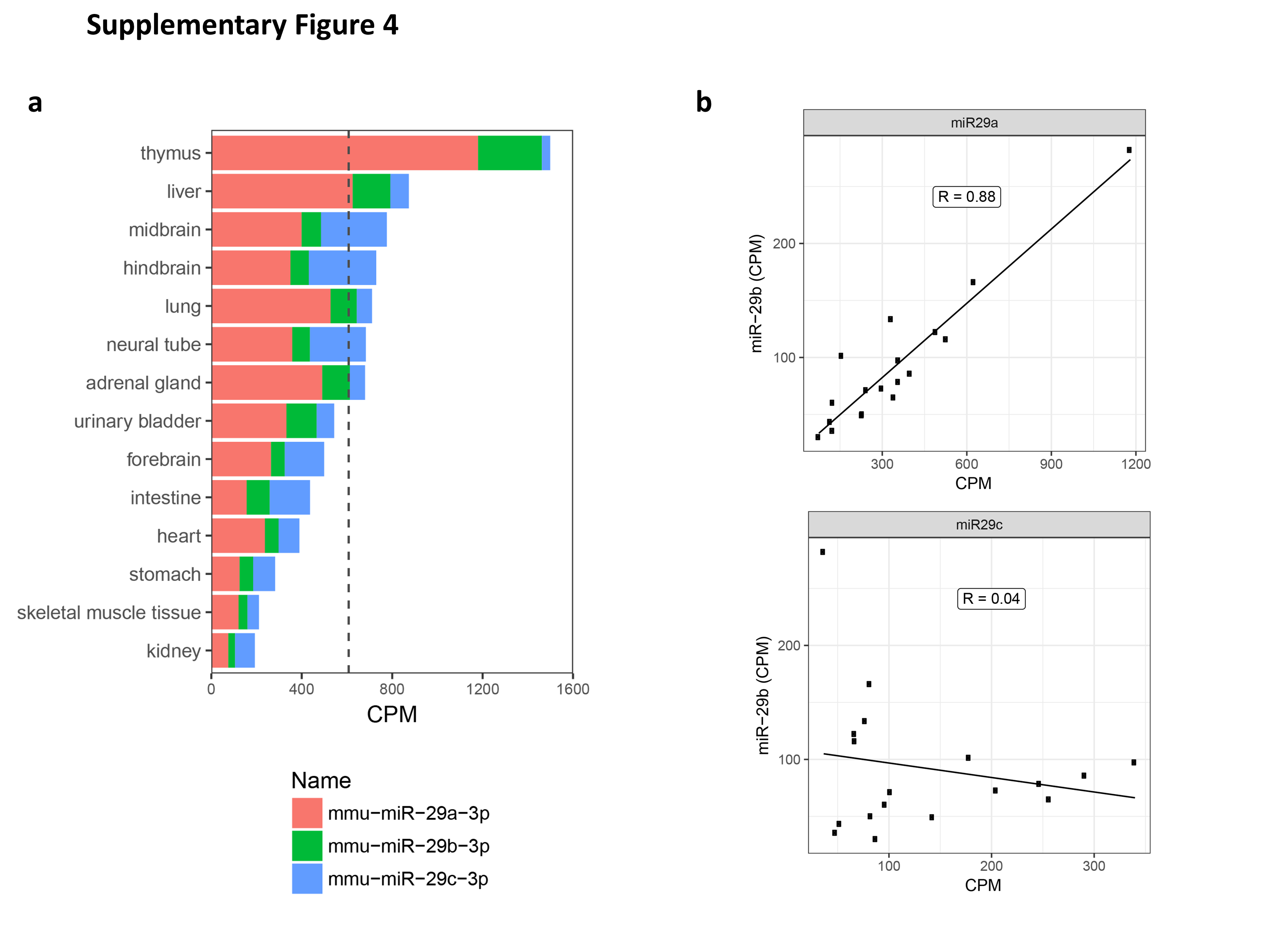

Supplement: S4 Fig — (A) miR-29 expression patterns from mouse postnatal day 0 tissues (ENCODE; average of duplicates if applicable). The dotted line indicates the mean expression value across all tissues. (B) Linear regression analysis between the expression of miR-29 family members from mouse postnatal day 0 tissues. Original raw data can be found in S1 Data file. CPM, counts per million; ENCODE, Encyclopedia of DNA Elements. (TIF) [file pbio.2006247.s004.TIF]

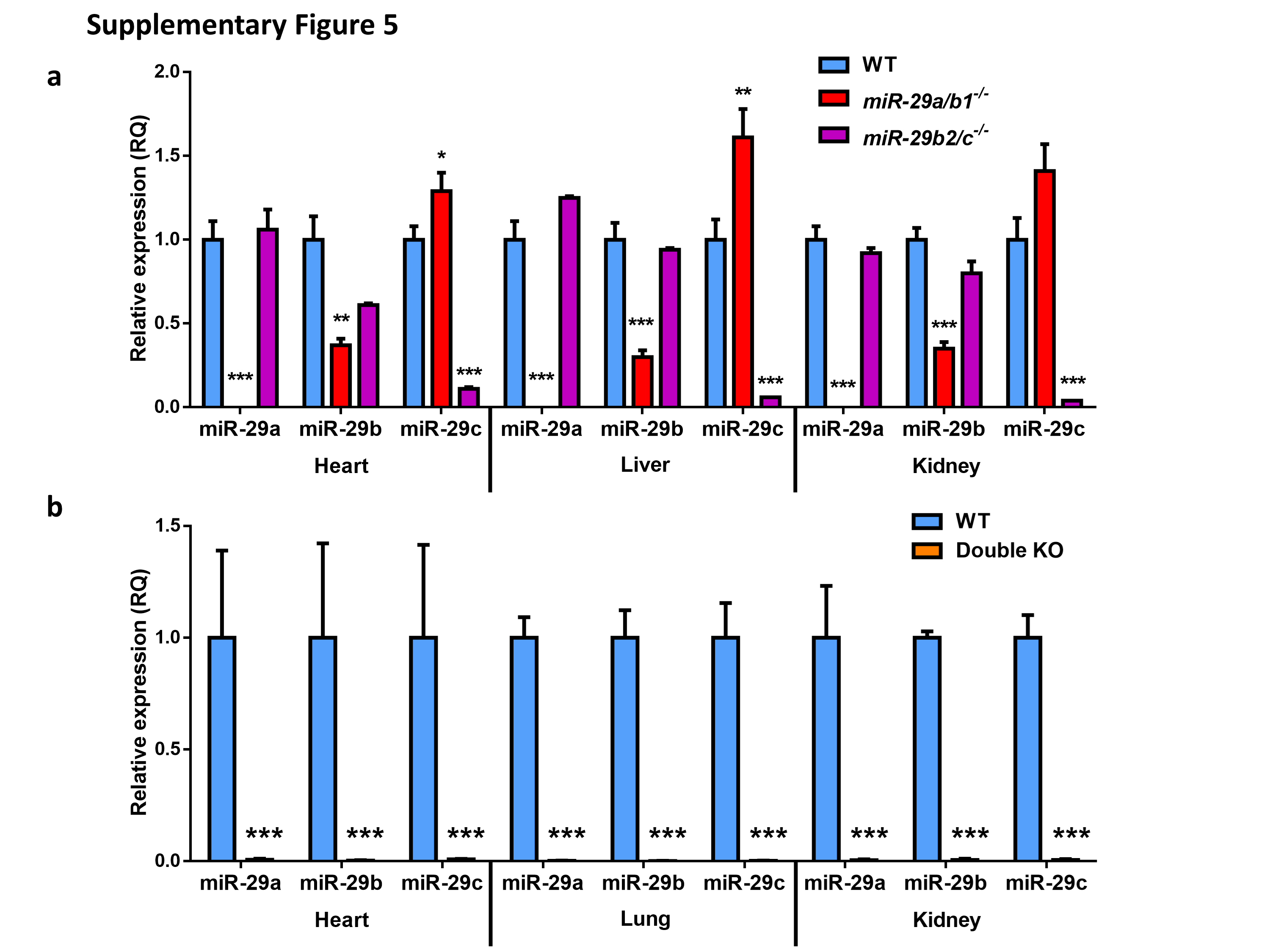

Supplement: S5 Fig — (A) Relative expression of miR-29 family members in heart, liver, and kidney samples from wild-type (n = 6), miR-29a/b1−/− (n = 3), and miR-29b2/c−/− (n = 3) mice. (B) Relative expression of miR-29 family members in heart, lung, and kidney samples from wild-type (n = 3) and double KO (n = 3) mice. Original raw data can be found in S1 Data file. KO, knock-out. (TIF) [file pbio.2006247.s005.TIF]

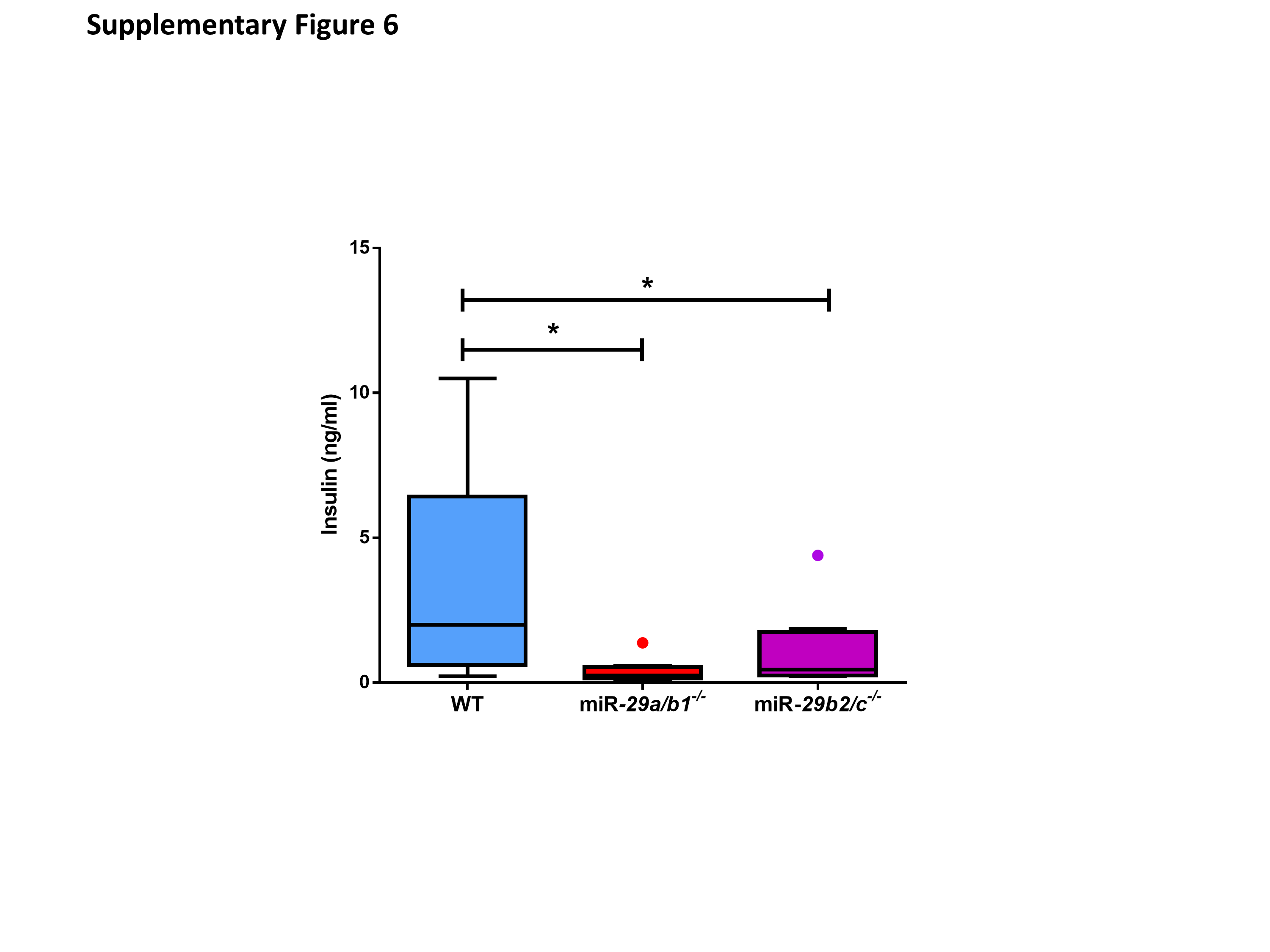

Supplement: S6 Fig — Levels of insulin measured by ELISA in serum from wild-type (n = 10), miR-29a/b1−/− (n = 8), and miR-29b2/c−/− (n = 8) mice. Original raw data can be found in S1 Data file. (TIF) [file pbio.2006247.s006.TIF]

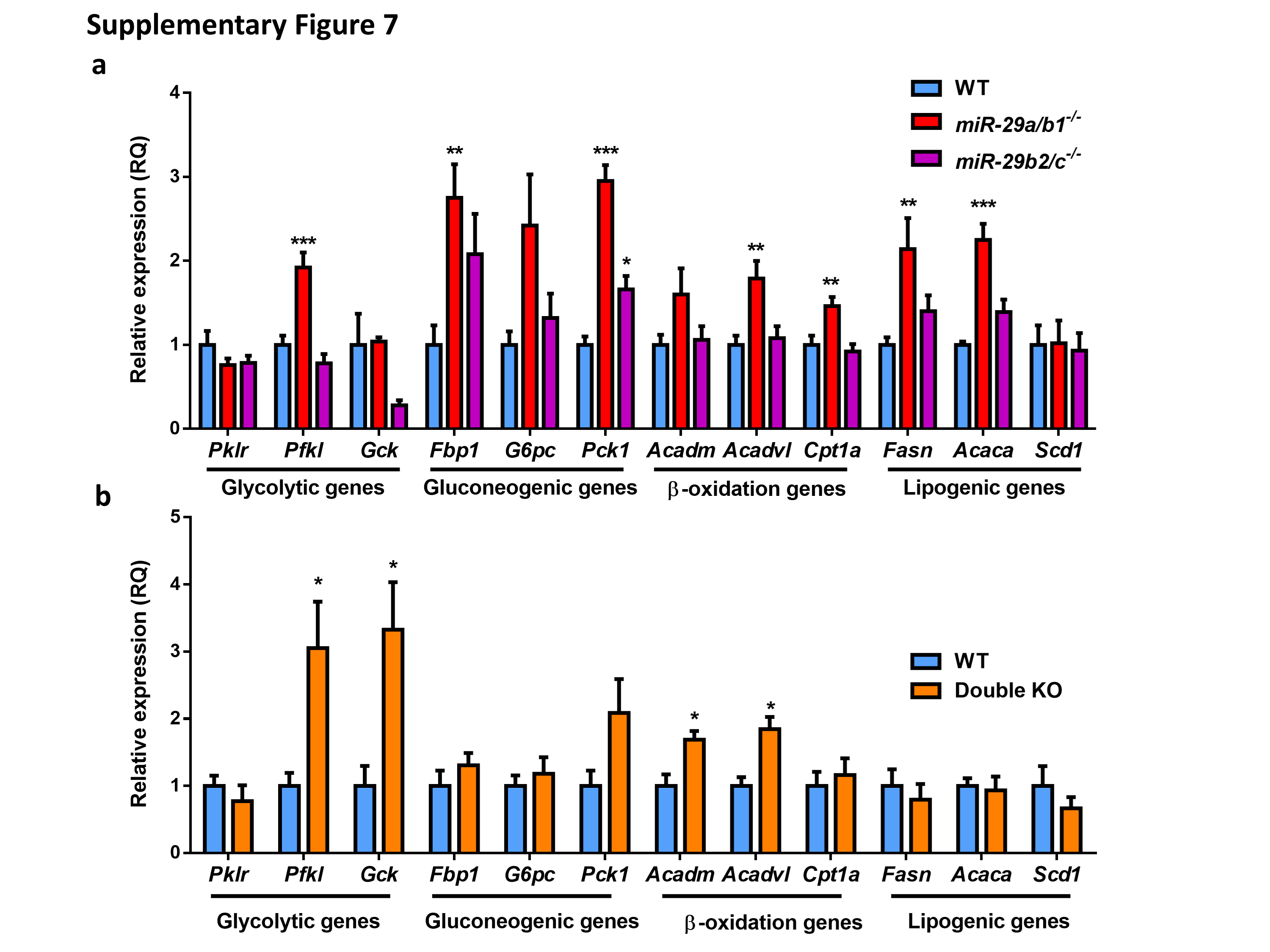

Supplement: S7 Fig — (A) Expression of key metabolic genes measured by RT-qPCR in livers from wild-type (n = 5), miR-29a/b1−/− (n = 5), and miR-29b2/c−/− (n = 4) mice. (B) Expression of key metabolic genes analyzed by RT-qPCR in livers from wild-type (n = 5) and double KO (n = 6) mice. Original raw data can be found in S1 Data file. RT-qPCR, quantitative reverse transcription PCR. (TIF) [file pbio.2006247.s007.TIF]

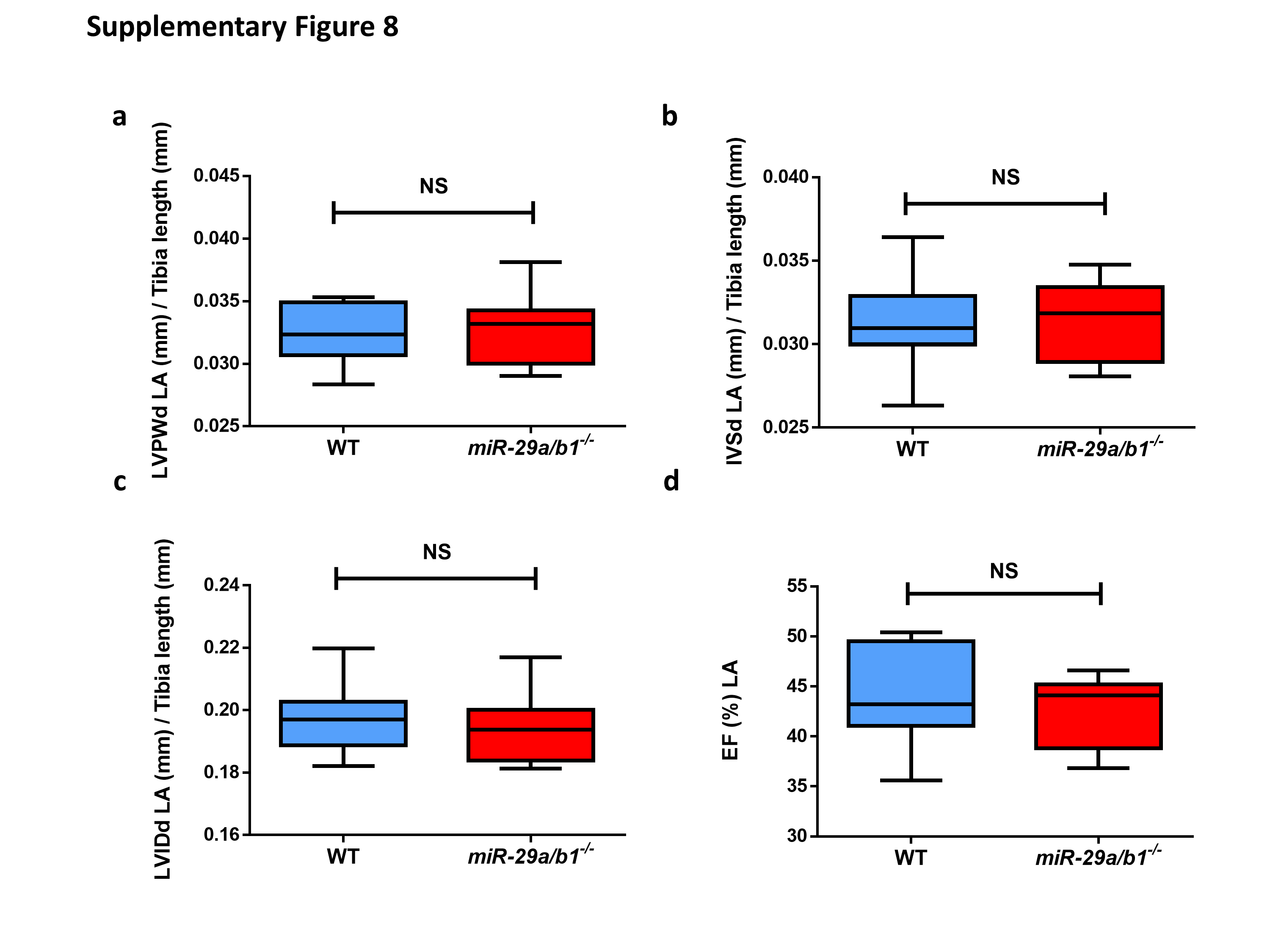

Supplement: S8 Fig — Quantification of structural parameters: (A) the left ventricular posterior wall (LVPW), (B) interventricular septum (IVS) thickness, and (C) left ventricular internal diameter (LVID), corrected by tibia length of wild-type (n = 10) and miR-29a/b1−/− (n = 9) mice. Quantification of functional parameters: (D) ejection fraction in wild-type (n = 10) and miR-29a/b1−/− (n = 8) mice. Original raw data can be found in S1 Data file. IVS, interventricular septum; LVID, left ventricular internal diameter; LVPW, left ventricular posterior wall. (TIF) [file pbio.2006247.s008.TIF]

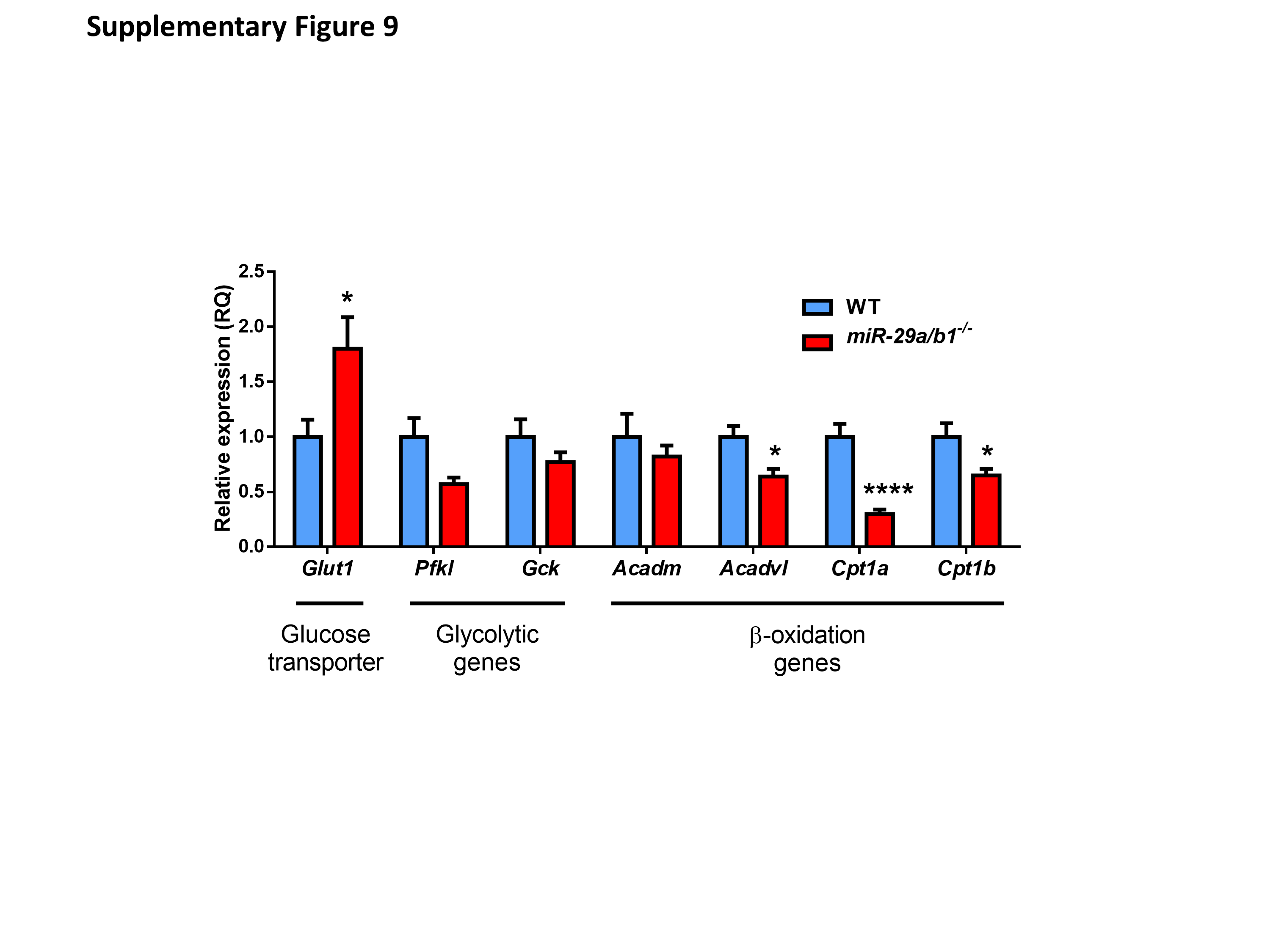

Supplement: S9 Fig — Expression of key metabolic genes measured by RT-qPCR in wild-type (n = 5) and miR-29a/b1−/− (n = 12) mice. Original raw data can be found in S1 Data file. RT-qPCR, quantitative reverse transcription PCR. (TIF) [file pbio.2006247.s009.TIF]

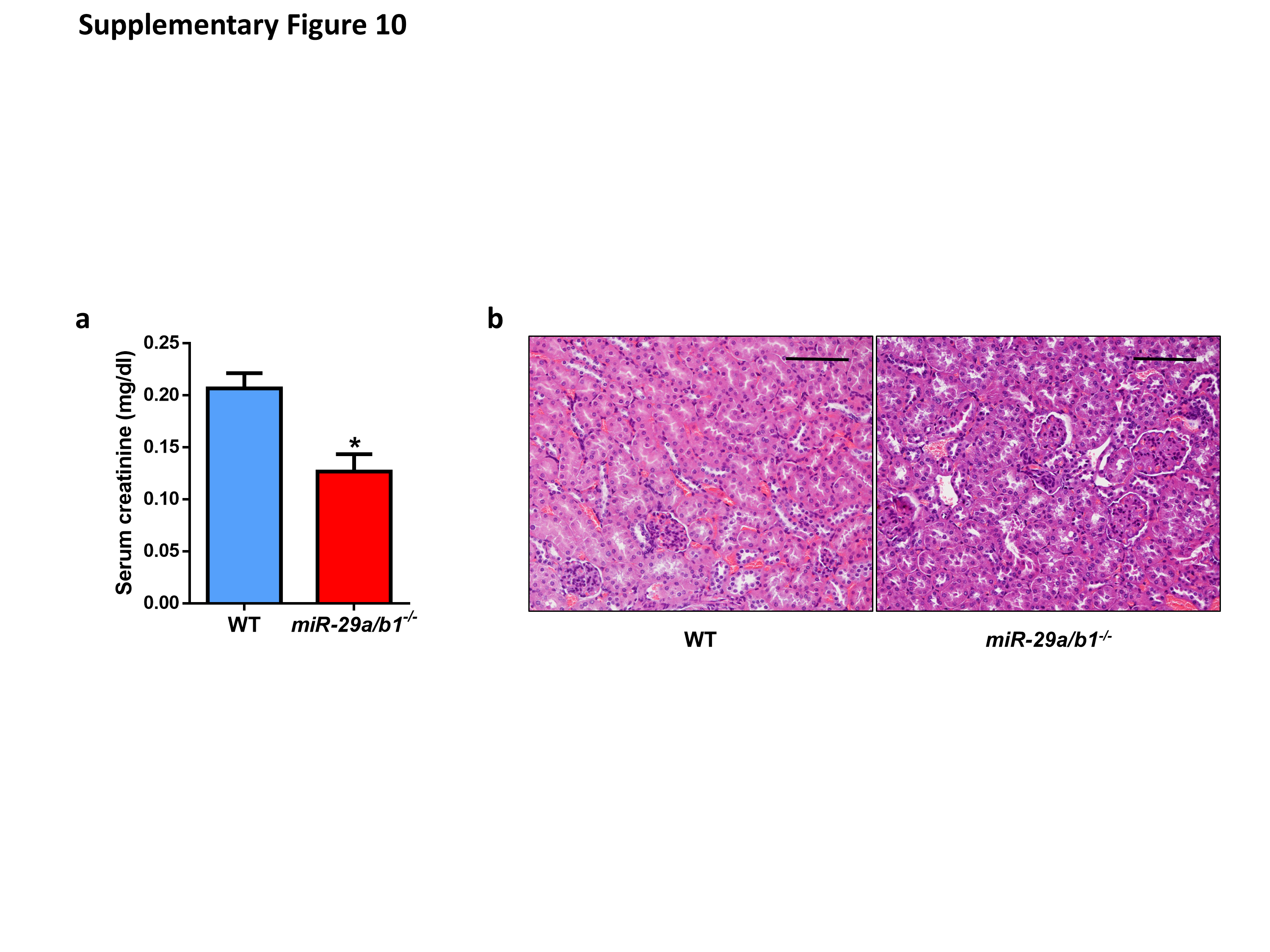

Supplement: S10 Fig — (A) Serum creatinine levels in wild-type (n = 3) and miR-29a/b1−/− (n = 3) mice. (B) HE sections of wild-type and miR-29a/b1−/− mice (original magnification: ×20, scale bar: 100 μm). Original raw data can be found in S1 Data file. HE, hematoxylin–eosin. (TIF) [file pbio.2006247.s010.TIF]

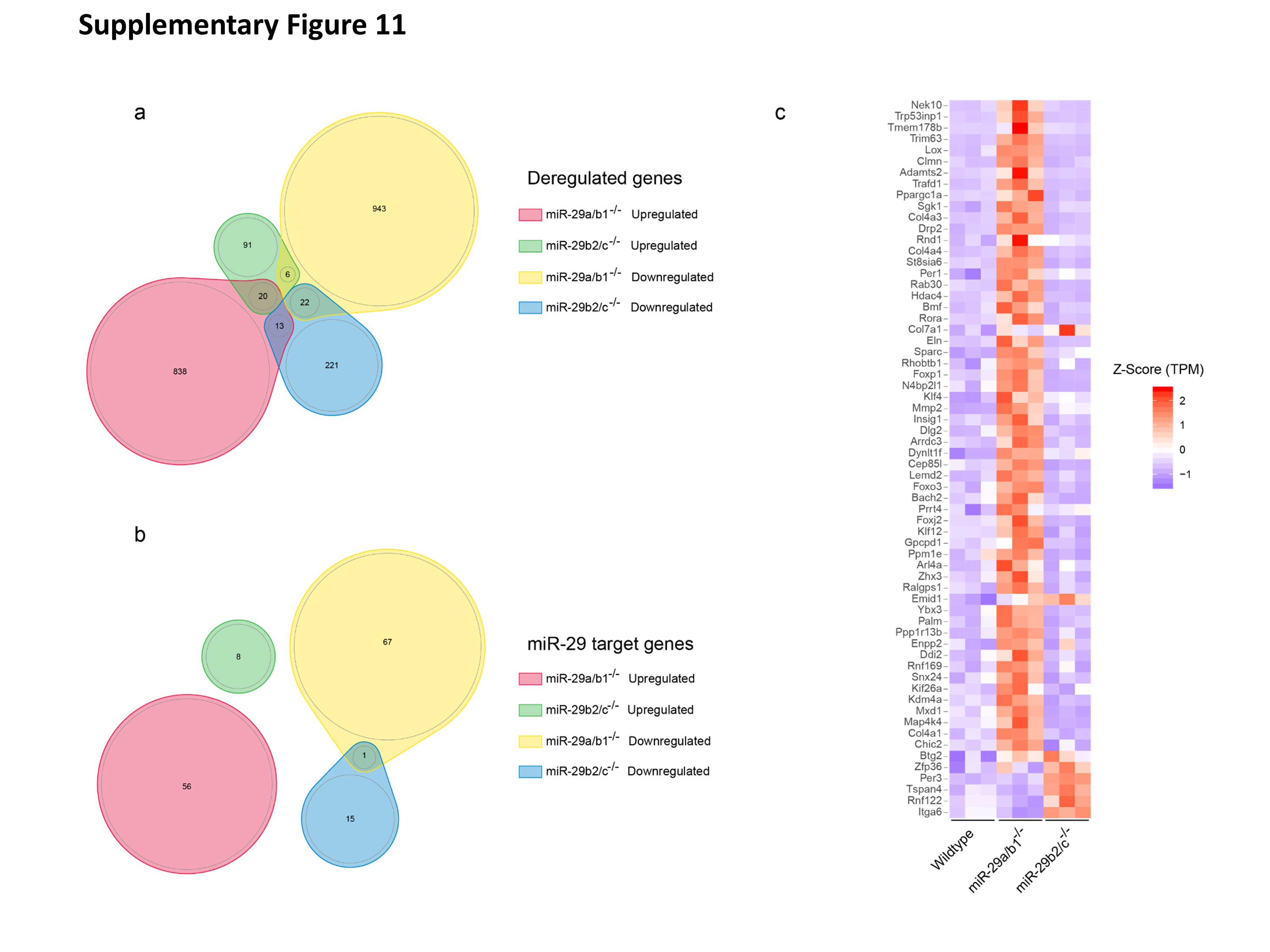

Supplement: S11 Fig — (A) Venn diagrams of differentially expressed genes (adjusted p-value < 0.01 and absolute log2 fold change > 0.8) in miR-29a/b1−/− and miR-29b2/c−/− mice compared with wild-type. (B) Venn diagram of differentially expressed miR-29–predicted target genes (adjusted p-value < 0.01 and absolute log2 fold change > 0.8) in miR-29a/b1−/− and miR-29b2/c−/− mice compared with wild-type. (C) Heat map plot of z-score normalized TPM of miR-29 predicted target genes significantly up-regulated in either in miR-29a/b1−/− and miR-29b2/c−/− mice compared with wild type. Original raw data can be found in S1 Data file. TPM, transcripts per million. (TIF) [file pbio.2006247.s011.TIF]

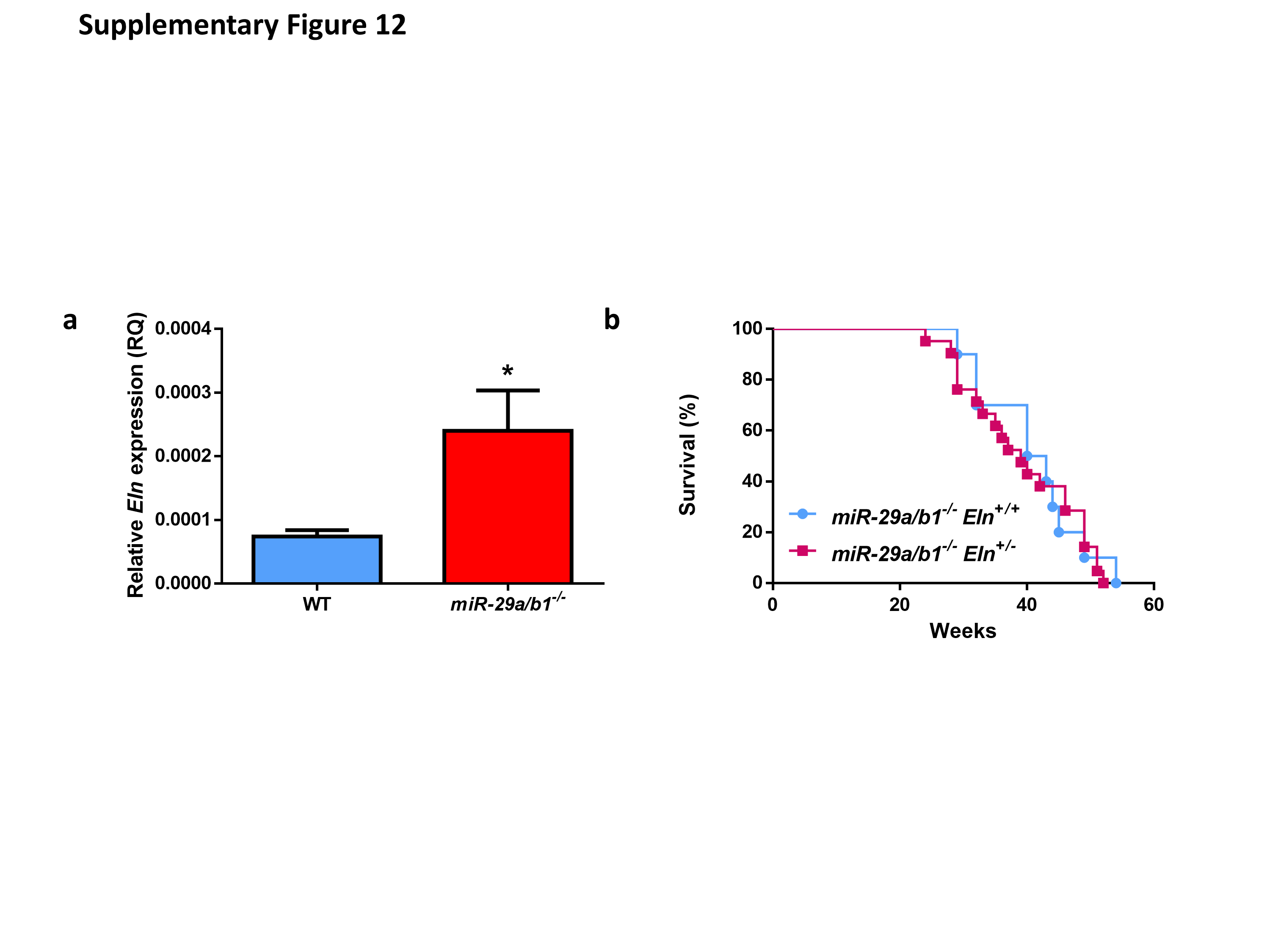

Supplement: S12 Fig — (A) Eln expression in hearts from wild-type (n = 4) and miR-29a/b1−/− (n = 4) mice. (B) Kaplan–Meier survival plot of miR-29a/b1−/− Eln+/+ (n = 10) and miR-29a/b1−/− Eln+/− (n = 21) mice. Original raw data can be found in S1 Data file. Eln, elastin. (TIF) [file pbio.2006247.s012.TIF]

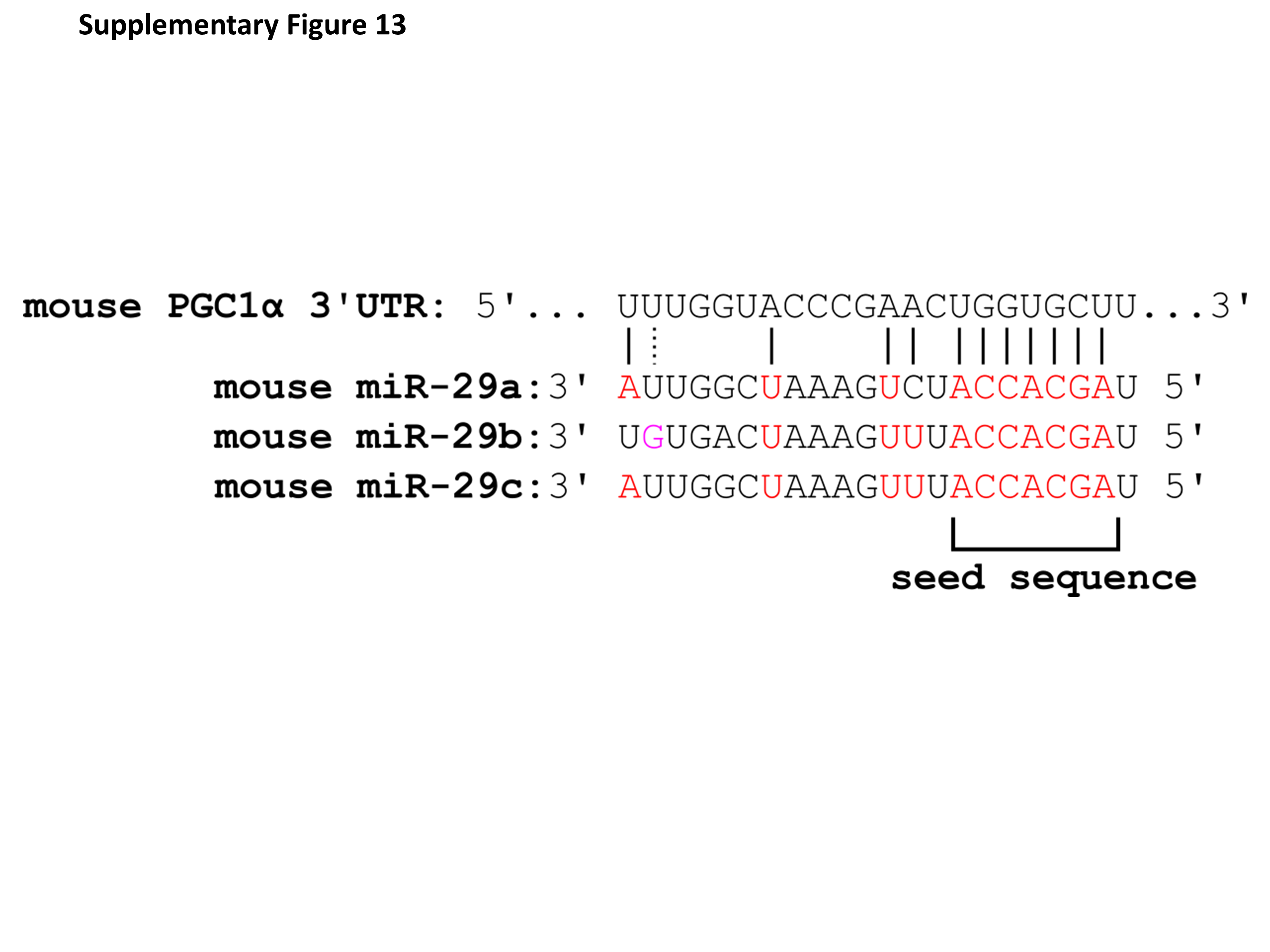

Supplement: S13 Fig — Sequence and putative miR-29–binding sites of PGC1α. All family members regulate PGC1α expression. Original raw data can be found in S1 Data file. (TIF) [file pbio.2006247.s013.TIF]

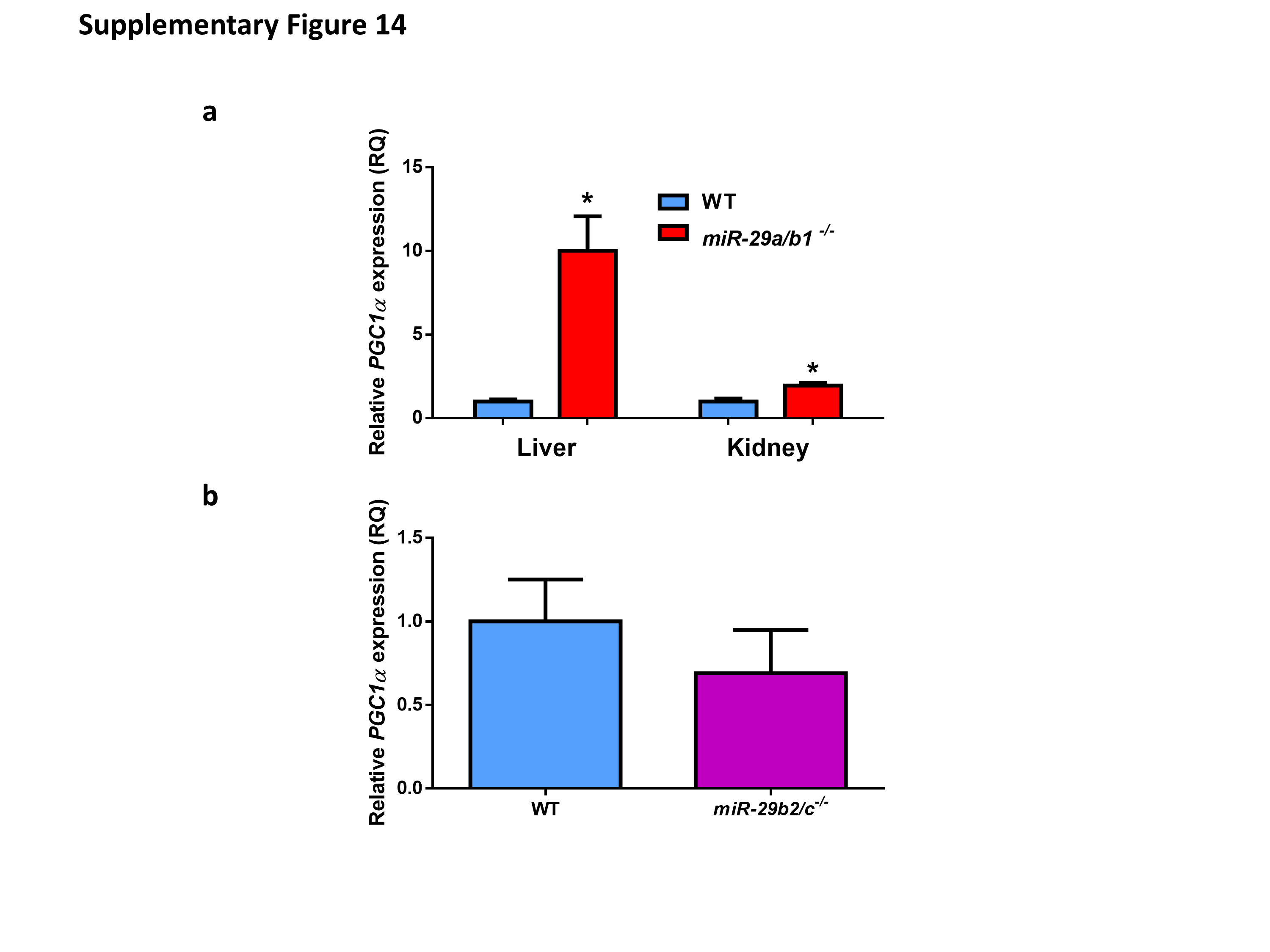

Supplement: S14 Fig — RT-PCRs of PGC1α in (A) liver and kidney tissues from wild-type (n = 4) and miR-29a/b1−/− (n = 4) mice and (B) hearts from wild-type (n = 3) and miR-29c/b2−/− (n = 3) mice. Original raw data can be found in S1 Data file. RT-PCR, quantitative reverse transcription PCR. (TIF) [file pbio.2006247.s014.TIF]

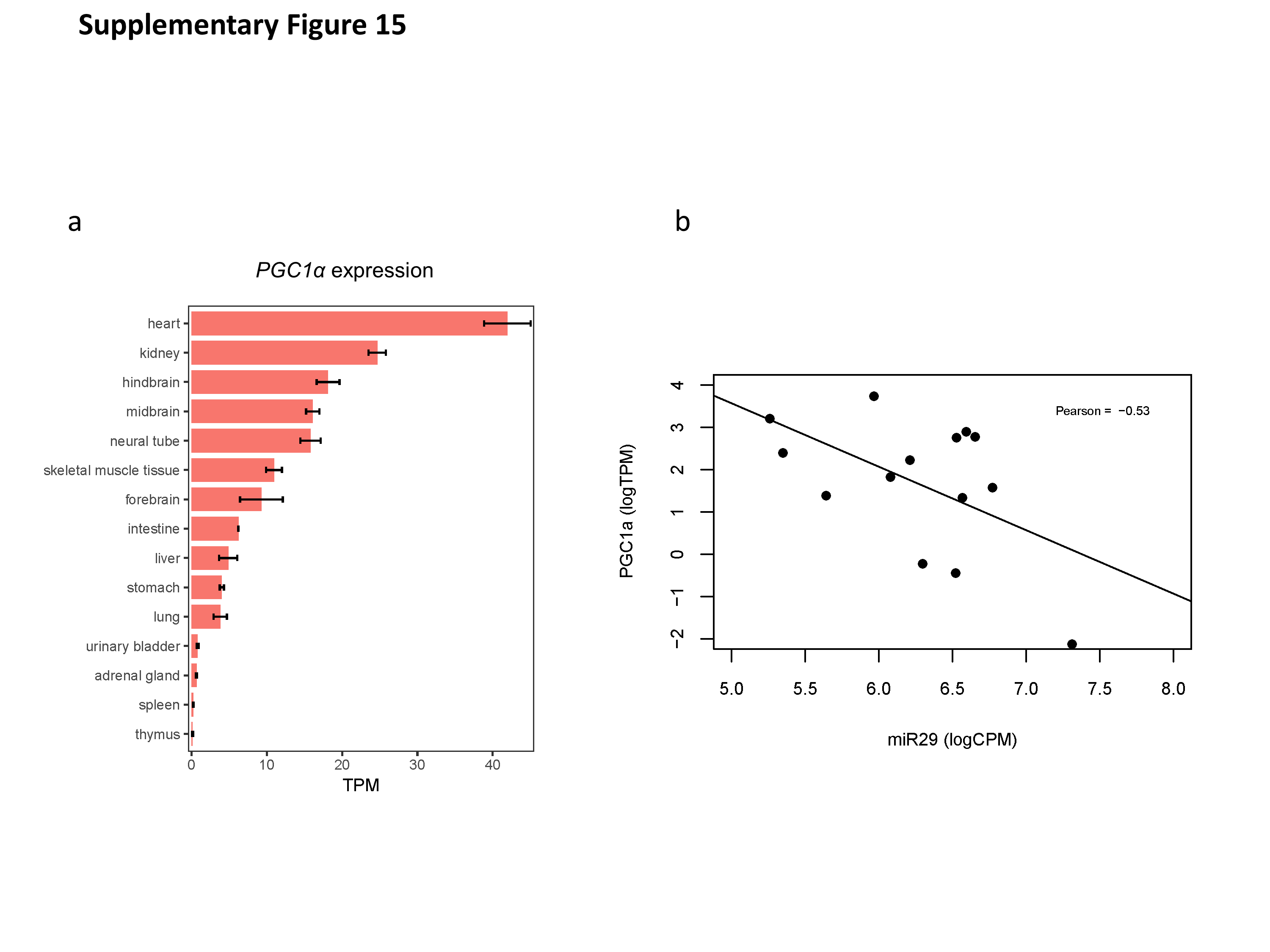

Supplement: S15 Fig — (A) PGC1α expression levels represented as transcripts per million (TPM) in different tissues from postnatal day 0 mice (average and standard error of the mean). (B) Pearson correlation between PGC1a and total miR-29 expression levels (sum of miR-29a, -b, and -c). Original raw data can be found in S1 Data file. CPM, counts per million; TPM, transcripts per million. (TIF) [file pbio.2006247.s015.TIF]

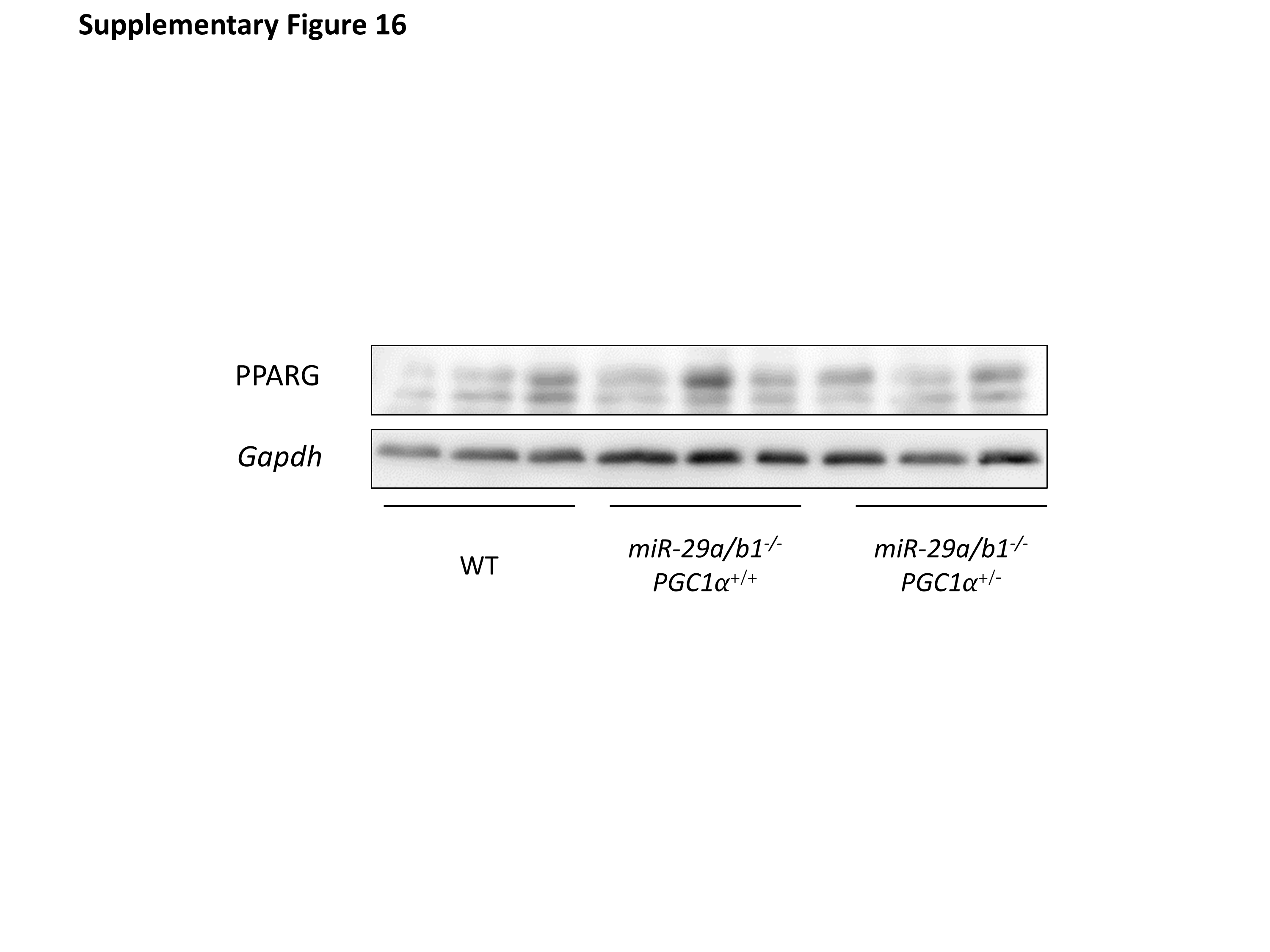

Supplement: S16 Fig — Western blot analysis using antibodies against Pparγ in protein extracts of hearts from wild-type (n = 3), miR-29a/b1−/− PGC1α+/+ (n = 3), and miR-29a/b1−/− PGC1α+/− (n = 3). Gapdh detection in the same blot was used as loading control. Original raw data can be found in S1 Data file. (TIF) [file pbio.2006247.s016.TIF]

Supplementary Figure 17

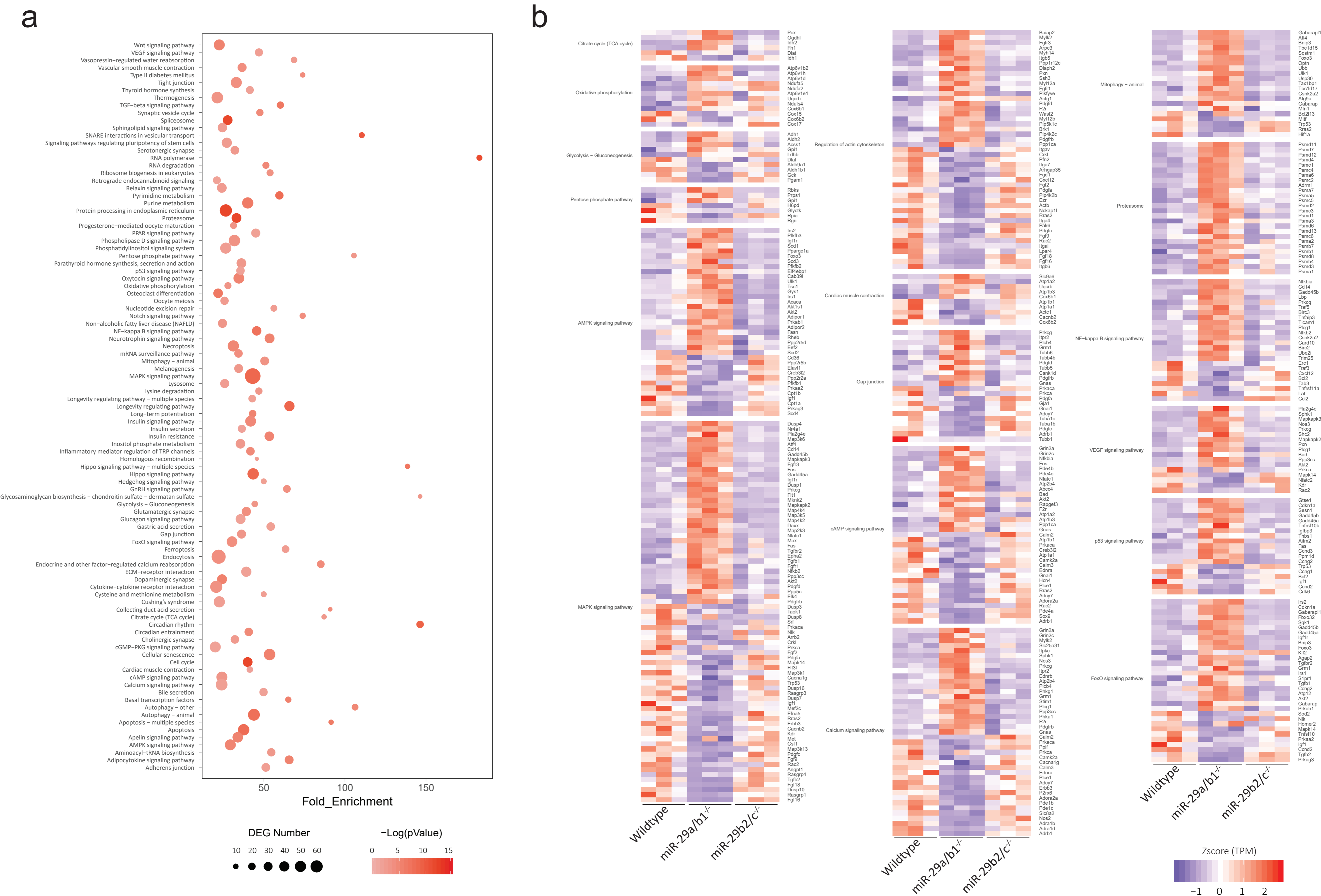

Supplement: S17 Fig — (A) “Bubble plot” showing pathways significantly enriched (adjusted enrichment p-value < 0.01; red color scale) in differentially expressed genes (DEG; size of the points) in miR-29a/b1−/− mice compared with wild-type mice. (B) Heat map plots of relevant pathways showing the z-score transformed TPM in hearts from wild-type, miR-29a/b1−/−, and miR-29c/b2−/− mice. Original raw data can be found in S1 Data file. DEG, differentially expressed gene; TPM, transcripts per million. (PDF) [file pbio.2006247.s017.pdf]

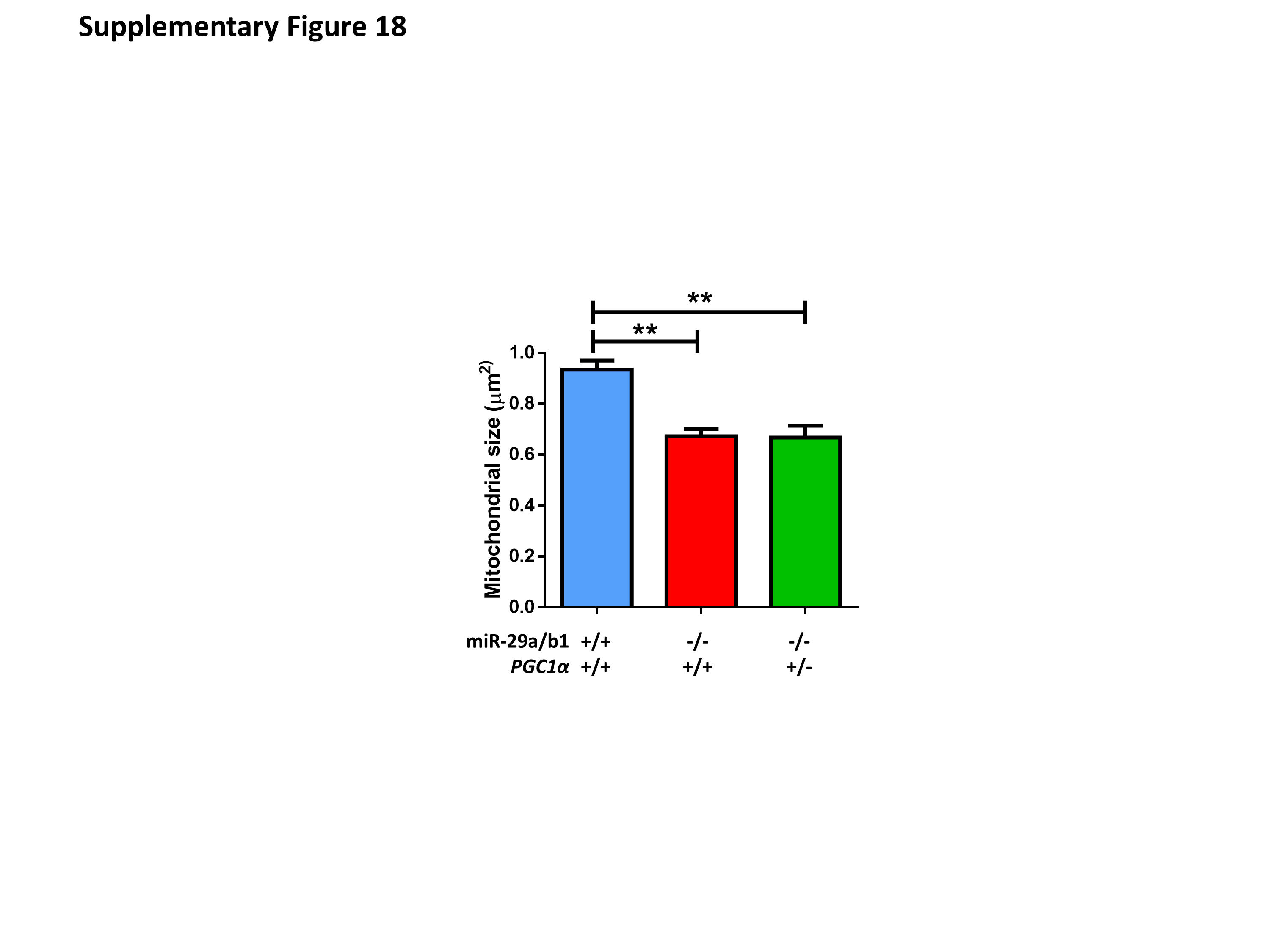

Supplement: S18 Fig — Quantification of mean mitochondrial size in wild-type (six photographs from two different mice), miR-29a/b1−/− PGC1α+/+ (22 photographs from four different mice), and miR-29a/b1−/− PGC1α+/− (22 photographs from three different mice) animals. Original raw data can be found in S1 Data file. (TIF) [file pbio.2006247.s018.TIF]

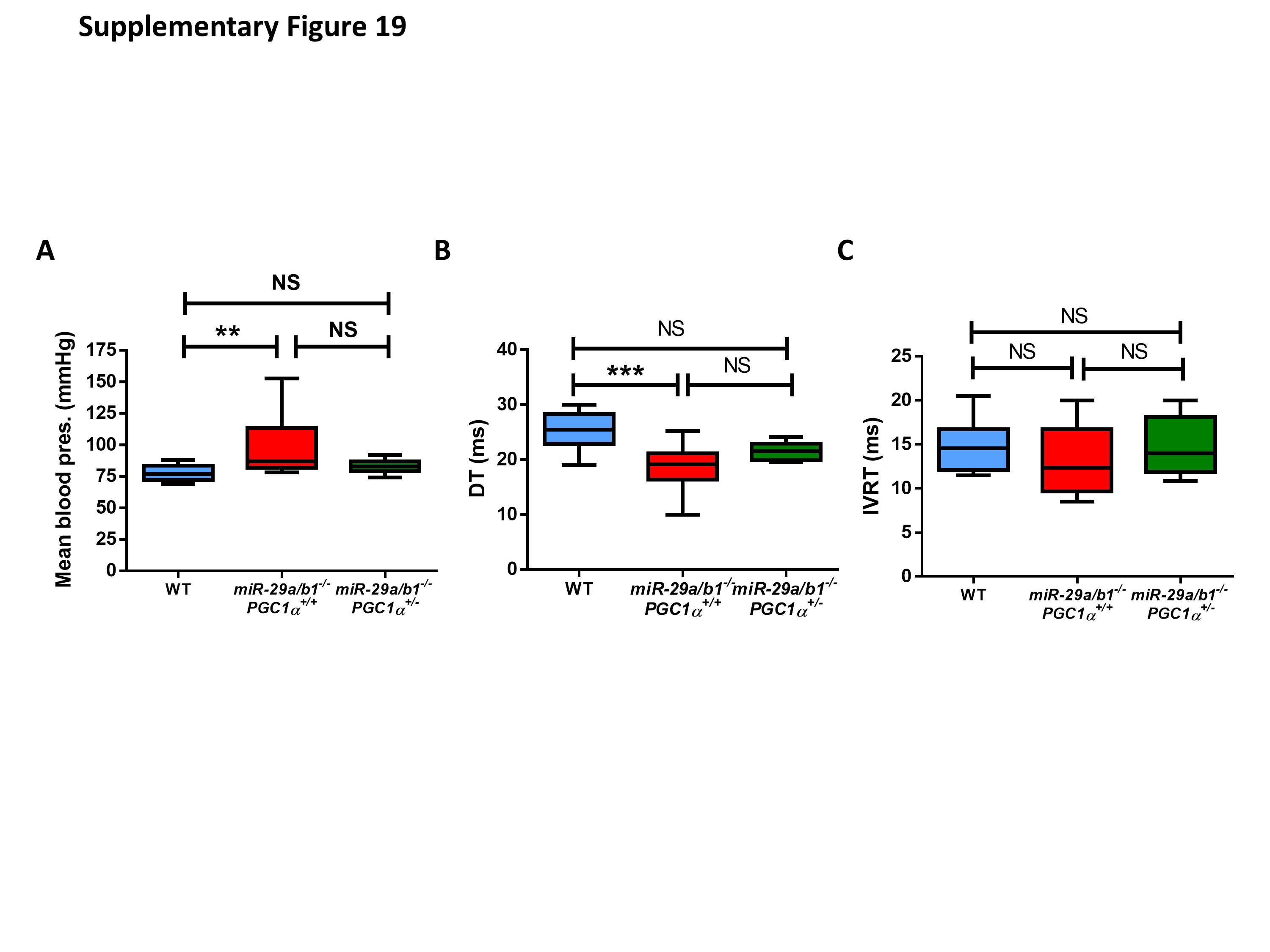

Supplement: S19 Fig — (A) Mean blood pressure values from wild-type (n = 10), miR-29a/b1−/− PGC1α+/+ (n = 8), and miR-29a/b1−/− PGC1α+/− (n = 6) mice. (B) Quantification of DT of early filling fraction in wild-type (n = 10), miR-29a/b1−/− PGC1α+/+ (n = 9), and miR-29a/b1−/− PGC1α+/− (n = 6) mice. (C) Quantification of IVRT in wild-type (n = 10), miR-29a/b1−/− PGC1α+/+ (n = 9), and miR-29a/b1−/− PGC1α+/− (n = 6) mice. Original raw data can be found in S1 Data file. DT, deceleration time; IVRT, isovolumetric relaxation time. (TIF) [file pbio.2006247.s019.TIF]
